# Lanthanide ion-doped upconversion nanoparticles for low-energy super-resolution applications

**DOI:** 10.1038/s41377-024-01547-6

**Published:** 2024-09-14

**Authors:** Simone Lamon, Haoyi Yu, Qiming Zhang, Min Gu

**Affiliations:** 1 School of Artificial Intelligence Science and Technology, University of Shanghai for Science and Technology, 200093 Shanghai, China; 2 Institute of Photonic Chips, University of Shanghai for Science and Technology, 200093 Shanghai, China

**Keywords:** Super-resolution microscopy, Nanoparticles

## Abstract

Energy-intensive technologies and high-precision research require energy-efficient techniques and materials. Lens-based optical microscopy technology is useful for low-energy applications in the life sciences and other fields of technology, but standard techniques cannot achieve applications at the nanoscale because of light diffraction. Far-field super-resolution techniques have broken beyond the light diffraction limit, enabling 3D applications down to the molecular scale and striving to reduce energy use. Typically targeted super-resolution techniques have achieved high resolution, but the high light intensity needed to outperform competing optical transitions in nanomaterials may result in photo-damage and high energy consumption. Great efforts have been made in the development of nanomaterials to improve the resolution and efficiency of these techniques toward low-energy super-resolution applications. Lanthanide ion-doped upconversion nanoparticles that exhibit multiple long-lived excited energy states and emit upconversion luminescence have enabled the development of targeted super-resolution techniques that need low-intensity light. The use of lanthanide ion-doped upconversion nanoparticles in these techniques for emerging low-energy super-resolution applications will have a significant impact on life sciences and other areas of technology. In this review, we describe the dynamics of lanthanide ion-doped upconversion nanoparticles for super-resolution under low-intensity light and their use in targeted super-resolution techniques. We highlight low-energy super-resolution applications of lanthanide ion-doped upconversion nanoparticles, as well as the related research directions and challenges. Our aim is to analyze targeted super-resolution techniques using lanthanide ion-doped upconversion nanoparticles, emphasizing fundamental mechanisms governing transitions in lanthanide ions to surpass the diffraction limit with low-intensity light, and exploring their implications for low-energy nanoscale applications.

## Introduction

Energy-intensive technologies such as data centers, illumination, transportation, and manufacturing require the development of sustainable processes enabled by energy-efficient techniques and materials^1–5^. Lower energy use in biology research and practices prevents damage to the samples and organisms being investigated while ensuring high precision and reliability, particularly for nanometer-scale studies^6–8^. Optical technology has been a driving force behind various developments that improve resolution and throughput while reducing energy consumption^9–11^. Among these, lens-based optical microscopy technology (Box 1) has shown to be useful for low-energy applications in the life sciences and other fields of technology such as light-enabled printing^12–14^, materials processing^15,16^, data storage^17–19^, and sensing^20^ among others. However, standard techniques cannot achieve applications at the nanoscale because of light diffraction^21^. Far-field super-resolution techniques^22,23^, including ‘stochastic’ and ‘deterministic’ (also known as ‘targeted’) techniques, have broken beyond the light diffraction limit, enabling 3D applications down to the molecular scale and striving to reduce energy use^24–29^.

Typically targeted super-resolution techniques use a focal excitation light intensity pattern featuring a strong spatial gradient to saturate optical transitions of nanomaterials to bright (‘activated’ or ‘ON’) and dark (‘inhibited’ or ‘OFF’) states^30^. These techniques have achieved high resolution, throughput, specificity, and selectivity, but the high light intensity needed to outperform competing optical transitions in nanomaterials may result in photo-damage and high energy consumption. Great efforts have been made in the development of nanomaterials with unique optical properties in order to improve the resolution and efficiency of targeted super-resolution techniques toward low-energy super-resolution applications^31–35^. The use of nanomaterials with excited energy states with long luminescence lifetimes can reduce the inhibition light intensity because the light intensity for saturation of inhibition is inversely proportional to the luminescence lifetime of the considered optical transitions^36,37^. Furthermore, the inhibition light intensity can be lowered using nanomaterials with a low light intensity threshold for saturation of inhibition^38,39^. A fundamentally different super-resolution approach is based on the use of nanomaterials that exhibit luminescence emission having a high order of non-linearity^40,41^. Such nanomaterials generate significant luminescence emission only at the spatial gradient maximum, that is from a region smaller than the size of the focal excitation light intensity pattern itself, resulting in enhanced resolution. This approach works without the need of inhibition light and shows promise for super-resolution applications using low-intensity light and simplified optical systems.

Among nanomaterials for targeted super-resolution techniques, lanthanide ion-doped upconversion nanoparticles (UCNPs) have shown unique physical, chemical, and optical properties^42–44^. Lanthanide ion-doped UCNPs are typically made of a host matrix, such as a rare earth oxide or fluoride, doped with trivalent lanthanide ions such as Yb^3+^, Er^3+^, and Tm^3+^^45,46^. These nanoparticles have distinctive electronic structures that feature a ladder-like arrangement of multiple excited energy states with long luminescence lifetimes ranging from microseconds to milliseconds or even longer. Lanthanide ion-doped UCNPs may convert photons with low photon energy like near-infrared (NIR) light into photons with higher photon energy like visible or ultraviolet (UV) light, producing upconversion luminescence (UCL) emission^47–51^. This process of photon upconversion of lanthanide ion-doped UCNPs necessitates excitation with low-intensity light and low laser energy density, commonly referred to as fluence. Furthermore, lanthanide ion-doped UCNPs have high brightness and photostability, and low toxicity^52–55^. These features make lanthanide ion-doped UCNPs attractive for use in many low-energy applications in the life sciences as well as other fields of technology because they use low-photon energy NIR excitation to produce higher-photon energy visible and UV UCL emission, enabling deep light penetration while necessitating low laser energy density to minimize both photo-damage and energy consumption^56–62^. Various reviews have explored the fundamental principles of generating, tuning, and enhancing UCL emission, and the synthesis and functionalization methodologies of lanthanide ion-doped UCNPs^63–67^. These studies also reported on the uses of lanthanide ion-doped UCNPs and UCNP-based nanomaterials in fields such as bioimaging, theranostics, photonics, and sensing^68–71^. However, the existing literature lacks an analysis of super-resolution techniques involving lanthanide ion-doped UCNPs, especially from a fundamental perspective. The discussion on the mechanisms governing transitions in lanthanide ions within UCNPs, allowing the surpassing of the fundamental diffraction limit with low-intensity light, is infrequent. Also, there is an absence of exploration into the potential implications of these mechanisms for low-energy applications at the nanometer scale.

**Fig. 1:**
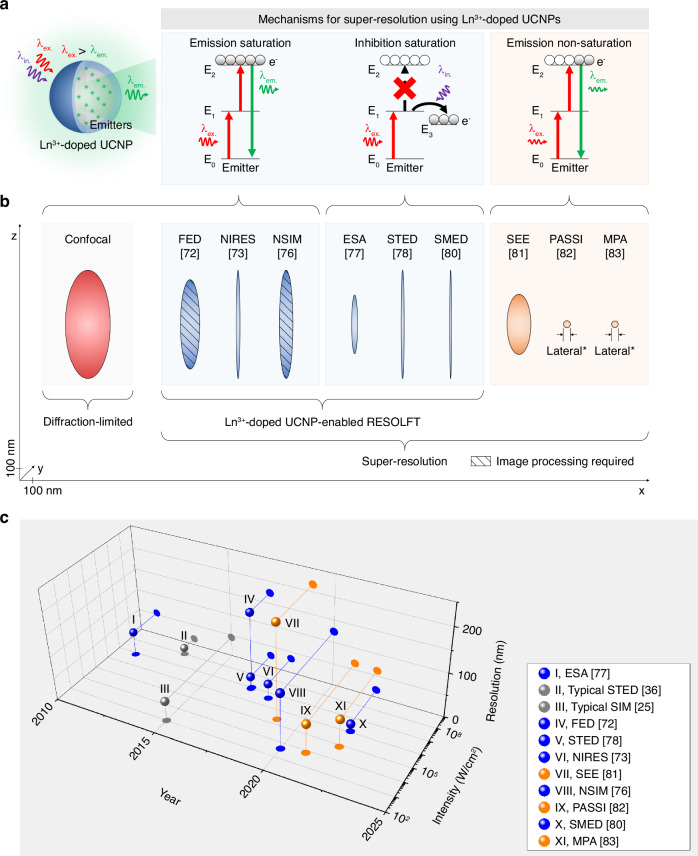
Overview of the working principles and features of targeted super-resolution techniques using lanthanide ion-doped UCNPs. **a** Schematic of the use of excitation and inhibition light to control optical transitions of lanthanide ion emitters of UCNPs for targeted super-resolution techniques based on UCL emission saturation, upconversion inhibition saturation, and UCL emission non-saturation. **b** Comparison of the 3D resolution achieved using different techniques using lanthanide ion-doped UCNPs. The ellipsoids represent the lateral (*x*,*y*) and axial (*z*) resolution of the stated techniques. Diffraction-limited confocal microscopy is shown in red, assuming the case of typical Yb^3+^/Tm^3+^-doped UCNPs that show UCL emission at 450 nm excited at 980 nm. Lanthanide ion-doped UCNP-enabled targeted super-resolution techniques based on UCL emission saturation, such as super-resolution FED microscopy^72^, NIRES nanoscopy^73^, and super-resolution NSIM^76^, or upconversion inhibition saturation, such as super-resolution ESA microscopy^77^, super-resolution STED microscopy^78^, and super-resolution SMED microscopy^80^, are shown in blue. Techniques based on UCL emission non-saturation, such as super-resolution SEE microscopy^81^, PASSI nanoscopy^82^, and MPA nanoscopy^83^, are shown in orange. **c** Timeline showing a comparison of the resolution and light intensity needed by different targeted super-resolution techniques including super-resolution STED microscopy^36^ and super-resolution SIM^25^ using typical fluorophores and luminescent nanomaterials, and techniques using lanthanide ion-doped UCNPs^72,73,76–78,80–83^. *For PASSI nanoscopy and MPA nanoscopy, only the value of the achieved lateral resolution is reported^82^^[,83^. λ_ex._ excitation wavelength, λ_em._ emission wavelength, λ_in._ inhibition wavelength, FED fluorescence emission difference, NIRES near-infrared emission saturation, NSIM nonlinear structured illumination microscopy, ESA excited-state absorption, STED stimulated emission depletion, SMED surface-migration emission depletion, SEE super-linear excitation-emission, PASSI photon-avalanche single-beam super-resolution imaging, MPA migrating photon avalanche

Lanthanide ion-doped UCNPs allowed for the development of targeted super-resolution techniques that control optical transitions of lanthanide ions of UCNPs using excitation and inhibition light to achieve UCL emission saturation^72–76^, upconversion inhibition saturation^77–80^, and UCL emission non-saturation^81–83^ (Fig. 1a). UCL emission saturation occurs through upconversion from the ground energy state to an emitting excited energy state for excitation light intensity to saturate upconversion. Upconversion inhibition saturation occurs through the depletion of an intermediate excited energy state to a non-emitting energy state for inhibition of light intensity to saturate depletion. Lanthanide ion-doped UCNP-enabled targeted super-resolution techniques based on these two working principles are inspired by typical ‘reversible saturable optical (fluorescence) transition (RESOLFT)’ techniques^22^. UCL emission non-saturation occurs for excitation light intensity lower than that to saturate upconversion. Targeted super-resolution techniques using lanthanide ion-doped UCNPs have allowed for super-resolution in both the lateral (*x*,*y*) and axial (*z*) directions, with a minimum lateral resolution of below 20 nm^80^ (Fig. 1b).

Targeted super-resolution techniques based on UCL emission non-saturation achieve super-resolution with a low light intensity of a few to tens of kW cm^−2^ and low energy density of ⁓10^−8^ J cm^−2^ to ⁓10^−10^ J cm^−2^^81–83^ (Fig. 1c). In comparison, targeted super-resolution techniques based on UCL emission saturation and upconversion inhibition saturation achieve super-resolution with a light intensity of up to ⁓10 MW cm^−2^ and an energy density of up to ⁓10^−5^ J cm^−2^^72–80^. Targeted super-resolution techniques based on typical fluorophores and luminescent nanomaterials achieve super-resolution with a light intensity of up to ⁓1–10 GW cm^−2^^36^ and an energy density of ⁓10^-4^ J cm^−2^^84^ to ⁓10^−2^ J cm^−2^^85^. Furthermore, lanthanide ion-doped UCNPs allow for achieving super-resolution using excitation and inhibition light from cheap and compact continuous-wave (CW) lasers, which is less damaging and reduces energy consumption, cost, and complexity of optical systems when compared to using light from expensive and bulky high-intensity pulsed lasers. The use of lanthanide ion-doped UCNPs in targeted super-resolution techniques for low-energy super-resolution applications will have a significant impact on life sciences and other areas of technology. However, there are still some challenges that need to be addressed before lanthanide ion-doped UCNPs can be widely employed, such as improving their spatial resolution, optimizing their optical properties, and further reducing light intensity.

In this review, we describe the dynamics of lanthanide ion-doped UCNPs for super-resolution under low-intensity light and their use in targeted super-resolution techniques. We highlight low-energy super-resolution applications of lanthanide ion-doped UCNPs, as well as the related research directions and challenges. Our goal is to offer the reader an analysis of super-resolution techniques using lanthanide ion-doped UCNPs, focusing on the fundamental aspects, including the mechanisms governing transitions in lanthanide ions within UCNPs to surpass the fundamental diffraction limit with low-intensity light. Also, we explore the potential implications of these mechanisms for low-energy applications at the nanometer scale.

## Dynamics for super-resolution using lanthanide ion-doped UCNPs

Lanthanide ion-doped UCNPs exhibit complex physical and chemical dynamics under light irradiation, including interactions between lanthanide ion dopants and the host matrix, energy transfer between different lanthanide ions, upconversion, and additional processes such as multi-phonon relaxations, cross-relaxation (CR) between excited energy states, and energy transfers to impurities and quenchers. The discussed phenomena and underlying mechanisms of lanthanide ion-doped UCNPs were previously examined, encompassing a review of both general and emerging strategies to exploit these phenomena and mitigate the related adverse effects^86^. Understanding and harnessing these dynamics of lanthanide ion-doped UCNPs is crucial to achieve super-resolution under low-intensity light for low-energy super-resolution applications. Because of their linear absorption, lanthanide ion-doped UCNPs have a larger NIR absorption cross-section than that of typical multi-photon fluorophores and luminescent nanomaterials^87,88^. The process of upconversion of lanthanide ion-doped UCNPs involves the sequential absorption of multiple photons, which is mediated by multiple long-lived intermediate excited energy states of lanthanide ions and allows for the use of excitation and inhibition light from low-intensity CW lasers. As a result, lanthanide ion-doped UCNPs avoid the critical need for simultaneous absorption of more than two photons from high-intensity pulsed lasers, resulting in several orders of magnitude lower light intensity threshold than that of typical multi-photon fluorophores and luminescent nanomaterials^89^.

**Fig. 2:**
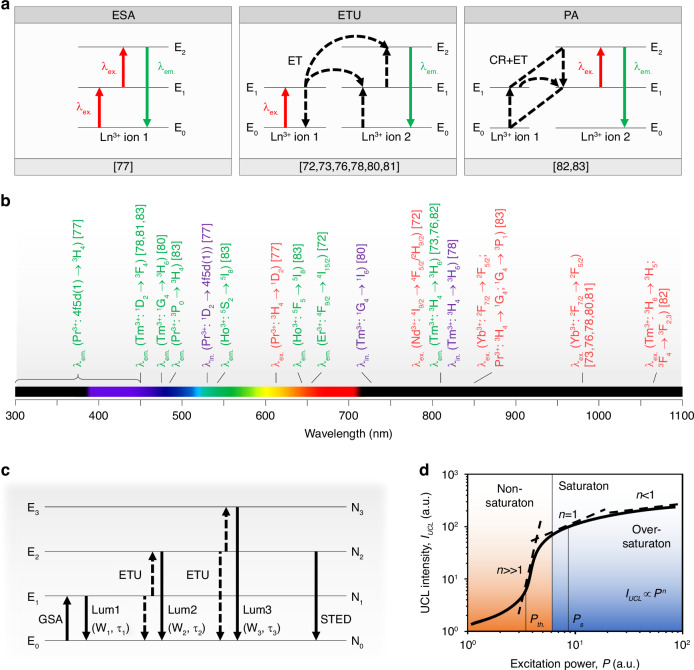
Analysis of the dynamics of optical transitions for super-resolution using lanthanide ion-doped UCNPs. **a** Schematic of the upconversion processes of ESA, ETU, and PA of targeted super-resolution techniques using lanthanide ion-doped UCNPs. **b** Summary of optical transitions of lanthanide ions of UCNPs, *λ*_*ex*._ (colored in red), *λ*_*in*._ (colored in purple), and *λ*_*em*_. (colored in green) of lanthanide ion-doped UCNPs used in the demonstrations of targeted super-resolution techniques^72–83^. **c** Schematic of a simplified four-state system used to describe the dynamics of optical transitions for super-resolution using lanthanide ion-doped UCNPs under excitation and inhibition light. **d** Schematic of the intensity saturation curve of lanthanide ion-doped UCNPs that shows the relationship between *I*_*UCL*_ and *P* under non-saturating and saturating light excitation. Lanthanide ion-doped UCNPs with highly super-linear^81^ and PA^82,83^ UCL emission show *n*»1 under non-saturating light excitation

Targeted super-resolution techniques using lanthanide ion-doped UCNPs are based on UCL emission through different mechanisms such as excited-state absorption (ESA), energy-transfer upconversion (ETU), and photon-avalanche (PA)^42,90,91^ (Fig. 2a). ESA involves excitation of a lanthanide ion through absorption in an already excited energy state. ETU involves energy transfer from one excited lanthanide ion called ‘sensitizer’, such as Yb^3+^ and Nd^3+^ ions, to a second neighboring lanthanide ion called ‘emitter’, such as Er^3+^ and Tm^3+^ ions, that results in excitation of the emitter to an excited state with higher energy and relaxation of the sensitizer to its ground energy state or excited state with lower energy. PA involves the excitation of a lanthanide ion through non-resonant ground-state absorption (GSA) and subsequent resonant ESA to an excited state with higher energy. CR between the excited lanthanide ion and a second neighboring lanthanide ion in its ground energy state results in both lanthanide ions occupying an excited energy state. The two lanthanide ions populate the excited state with higher energy in the first lanthanide ion to further initiate CR. This process exponentially increases the electronic population of the excited state with higher energy in the first lanthanide ion through ESA, resulting in high-intensity UCL emission.

Targeted super-resolution techniques using lanthanide ion-doped UCNPs have overcome the light diffraction limit in 3D using different excitation and inhibition light wavelengths to control optical transitions of lanthanide ion-doped UCNPs that show upconversion emission and inhibition from a wide range of lanthanide ions (Fig. 2b). A simplified model describing the dynamics of lanthanide ion-doped UCNPs for super-resolution under low-intensity light is reported on the basis of a four-state system with a ground energy state *E*_*0*_ and three excited energy states *E*_*i*_ with *i* = 1–3 (Fig. 2c). It is assumed that the population density in the ground energy state *N*_*0*_ is constant, the system is pumped under CW light excitation through GSA, upconversion is achieved through ETU with parameters *W*_*i*_ for *E*_*i*_, *E*_*i*_ has luminescence lifetime *τ*_*i*_ and decays with rate constant *A*_*i*_ = *τ*_*i*_^−1^ either to the next lower excited energy state or to the ground energy state, and STED is considered as the mechanism for upconversion inhibition. The rate equations that describe upconversion excitation and inhibition in this system are:

$${{\rm{d}}N}_{1}/{{\rm{d}}}_{t}={\rho }_{p}{\sigma }_{p}{N}_{0}-2{W}_{1}{N}_{1}{N}_{1}-{W}_{2}{N}_{1}{N}_{2}-{A}_{1}{N}_{1}+{A}_{2}{N}_{2}$$

$$\displaystyle{\rm{d}}{N}_2/{\rm{d}}{t}=\rho {_{STED}}\sigma {_{STED}}({N}_{2}-{N}_{0})+{W}_{1}{N}_{1}{N}_{1}-{W}_{2}{N}_{1}{N}_{2}-{A}_{2}{N}_{2}+{A}_{3}{N}_{3}$$

$${{\rm{d}}N}_{3}/{{\rm{d}}}_{t}={W}_{2}{N}_{1}{N}_{2}-{A}_{3}{N}_{3}$$

Where *N*_*i*_ is the population density in *E*_*i*_ satisfying *N*_*0*_ + *N*_*1*_ + *N*_*2*_ + *N*_*3*_ = 1, *ρ*_*p*_ is the pump constant, *σ*_*p*_ is the absorption cross section from *E*_*0*_ at the pump wavelength, *ρ*_*STED*_ is the STED constant, *σ*_*STED*_ is the STED cross-section from *E*_*2*_ at the STED wavelength. *ρ*_*p*_ = (*λ*_*p*_/*hcπw*^2^_*p*_)*P*_*pump*_, where *λ*_*p*_ is the pump wavelength, *h* is Planck’s constant, *c* is the light speed, and *w*_*p*_ is the pump beam radius. *ρ*_*STED*_ = (*λ*_*STED*_/*hcπw*^2^_*STED*_)*P*_*STED*_, where *λ*_*STED*_ is the STED wavelength, and *w*_*STED*_ is the STED beam radius.

For upconversion excitation, the numerical solution of these rate equations gives that the *I*_*UCL*_ that is excited by *n* photons that are absorbed sequentially depends on the pump light power *P* that is absorbed as *I*_*UCL*_ ∝ *P*^*i*^^92^. *i* ranges from *i* = *n* > 1 for infinitely small upconversion rates, that is the intermediate excited energy states depletion proceeds preferentially via luminescence emission rather than upconversion, to *i* ≤ 1 for infinitely large upconversion rates, that is the depletion of intermediate excited energy states proceeds preferentially via upconversion rather than luminescence emission. This dependency suggests that the optical transitions of lanthanide ions of UCNPs are saturated as the excitation light intensity increases. Upconversion saturation typically occurs under low-intensity light excitation in the range from tens to hundreds of kW cm^−2^. For upconversion excitation and inhibition, the electronic population in *E*_*2*_ is depleted to *E*_*0*_ through STED. The intensity ratio of UCL emission for the STED light beam switched ON and OFF, that is called ‘depletion ratio’, increases by rising the STED light beam intensity to saturate the depletion of *E*_*2*_ to *E*_*0*_ through stimulated emission. In principle, longer luminescence lifetimes enable to reduce the intensity of the STED light beam. Because the 4f-4f transitions of lanthanide ions of UCNPs are forbidden, they have long luminescence lifetimes ranging from tens of microseconds to milliseconds or longer. As a result, lanthanide ion-doped UCNPs allow for mitigating the square root law which underpins resolution scaling in RESOLFT techniques.

The intensity saturation curve of lanthanide ion-doped UCNPs shows the relationship between *I*_*UCL*_ and *P* (Fig. 2d). In a log-log plot, the slope of such a curve at a certain *P* corresponds to the value of *n*, which typically indicates the order of non-linearity of upconversion. For low *P*, lanthanide ion-doped UCNPs show weak *I*_*UCL*_. An increase of *P* under non-saturating light excitation results in an increase of *I*_*UCL*_ with a super-linear regime. For lanthanide ion-doped UCNPs that show highly super-linear^81^ and PA^82,83^ UCL emissions, the slope is of *n* >> 1. When the excitation light beam intensity is within the highly super-linear range of the intensity saturation curve of lanthanide ion-doped UCNPs, only the central and most intense section of the excitation light beam produces a strong UCL emission. As a result, the UCL emission profile of lanthanide ion-doped UCNPs is narrower and steeper than the excitation light beam profile, facilitating super-resolution under low-intensity light excitation. PA UCNPs show a clear excitation-power threshold *P*_*th*._ above which a large non-linear increase in the excited-state population and UCL emission is observed with a steep slope of their intensity saturation curve. Further increase of *P* under saturating light excitation results in a linear regime with a slope of *n* = 1 for *P* = *P*_*s*_, a sub-linear regime with a slope of *n* < 1 for *P* > *P*_*s*_, and then over-saturation regime.

## Targeted super-resolution techniques using lanthanide ion-doped UCNPs

### Targeted super-resolution techniques based on UCL emission saturation

#### Super-resolution FED microscopy

Super-resolution FED microscopy relies on the intensity difference between two images called ‘solid’ and ‘donut’ images that are acquired by scanning the sample with solid and donut excitation light patterns, respectively^93^. Applying fluorescence saturation enhances the resolution capability of super-resolution FED microscopy^94^. This technique provides a straightforward pathway to super-resolution imaging without the need for high-depleting laser intensities. Additionally, it is independent to the species of agents, underscoring its versatility and promising potential. However, conventional fluorescent dyes used in super-resolution FED microscopy are prone to photobleaching, limiting their suitability for extended imaging, while high illumination intensities exacerbate this issue and pose risks of sample damage. Leveraging lanthanide ion-doped UCNPs shows great promise for super-resolution FED microscopy due to their outstanding photostability and efficiency of UCL emission when excited by NIR CW lasers.

**Fig. 3:**
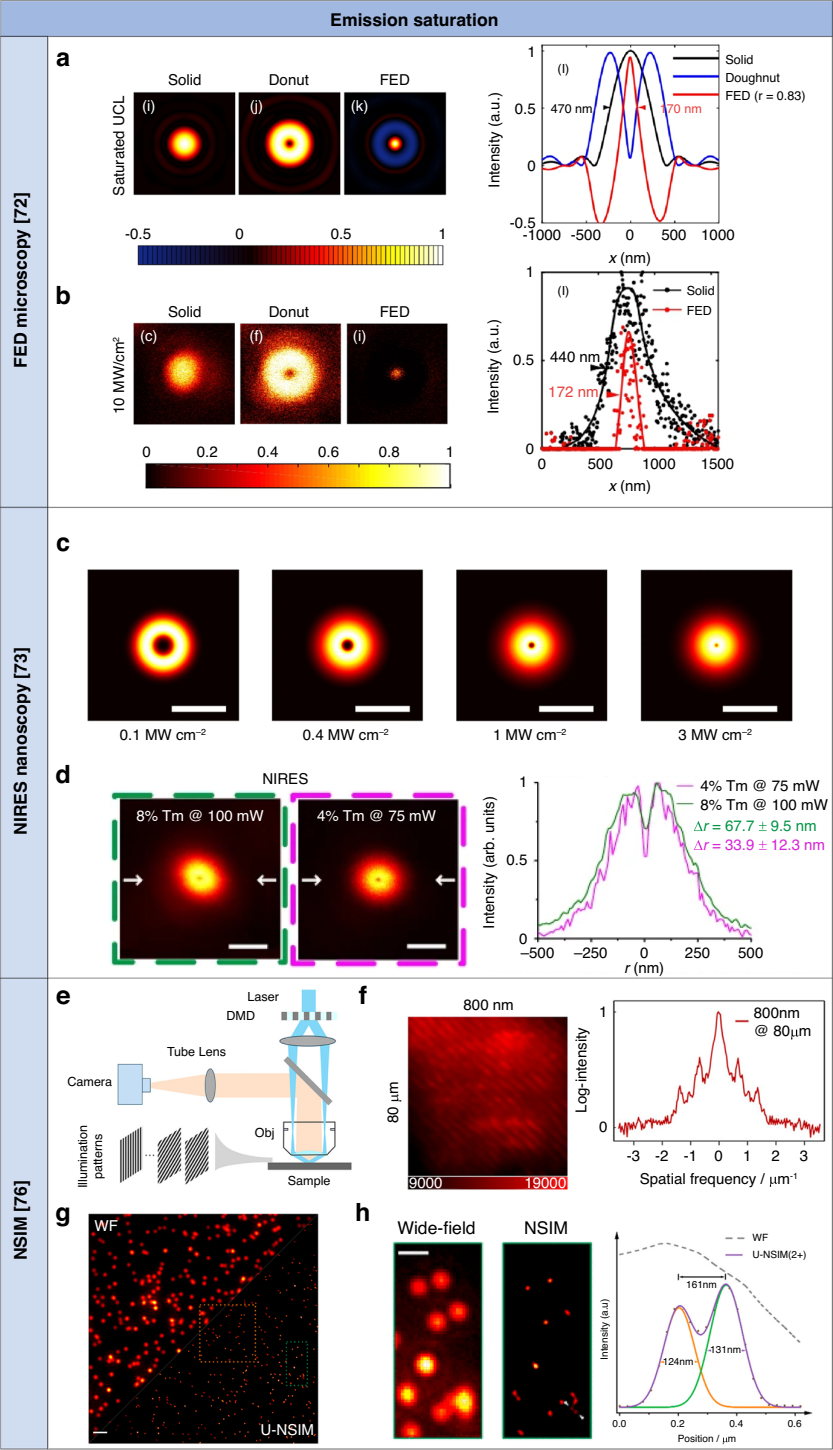
Summary of targeted super-resolution techniques based on UCL emission saturation of lanthanide ion-doped UCNPs. **a** Simulated solid, donut, and super-resolution FED PSFs, and corresponding intensity profiles of saturated UCL emission of lanthanide ion-doped UCNPs. Adapted with permission from ref. ^72^ Copyright 2017 Optical Society of America^72^. **b** Solid, donut, and super-resolution FED microscopy imaging, and corresponding intensity profiles of saturated UCL emission of lanthanide ion-doped UCNPs. Image size: 1.56 μm. Adapted with permission from ref. ^72^ Copyright 2017 Optical Society of America^72^. **c** Simulated ‘negative’ contrast images of cross-section profiles of the saturated UCL emission of a lanthanide ion-doped UCNP used in NIRES nanoscopy at different excitation intensity. Scale bar: 500 nm. Reproduced with permission from ref. ^73^, CC BY 4.0. Copyright 2018 Springer Nature Limited^73^. **d** NIRES nanoscopy imaging and corresponding intensity profiles of lanthanide ion-doped UCNPs with different doping. Scale bar: 500 nm. Adapted with permission from ref. ^73^, CC BY 4.0. Copyright 2018 Springer Nature Limited^73^. **e** Schematic of the super-resolution NSIM setup using lanthanide ion-doped UCNPs. Reproduced with permission from ref. ^76^. Copyright 2020 American Chemical Society^76^. **f** UCL emission imaging of lanthanide ion-doped UCNPs with 80 μm thick brain tissues under sinusoidal structured excitation and line profile of Fourier spectrum on a logarithmic scale of the diagonal cross-section profiles. Adapted with permission from ref. ^76^. Copyright 2020 American Chemical Society^76^. **g** Wide-field and super-resolution NSIM imaging of lanthanide ion-doped UCNPs. Scale bar: 2 μm. Reproduced with permission from ref. ^76^. Copyright 2020 American Chemical Society^76^. **h** Comparison imaging results of the green framed area in (**g**), and corresponding intensity profiles of lanthanide ion-doped UCNPs. Scale bar: 1 μm. Reproduced with permission from ref. ^76^. Copyright 2020 American Chemical Society^76^

Super-resolution FED microscopy was achieved based on UCL emission saturation using core-shell Nd^3+^/Yb^3+^/Er^3+^-doped UCNPs^72^. The Nd^3+^ ions in the shell absorbed energy from 808-nm excitation photons, which were then transferred to the Yb^3+^ ions in the core. From there, the energy was further transmitted from Yb^3+^ to Er^3+^ ions, enabling the UCL emission of green and red light. Solid, donut, and super-resolution FED point spread functions (PSFs) were simulated for saturated UCL emission of these lanthanide ion-doped UCNPs under 808-nm laser excitation at an intensity of 10 MW cm^−2^ (Fig. 3a). The corresponding super-resolution FED PSF was obtained with a subtraction factor set to 0.83. Subsequently, a single lanthanide ion-doped UCNP was imaged using a solid light pattern at 10 MW cm^−2^ and a donut light pattern at the same intensity, and detecting UCL emission at 655 nm (Fig. 3b). The lateral resolution was 172 nm for saturated UCL emission under sequential solid and donut light pattern excitation with a light intensity of 10 MW cm^−2^ and with a subtraction factor of 0.83. The photostability of lanthanide ion-doped UCNPs for super-resolution FED microscopy was examined under prolonged laser exposure lasting tens of minutes, with a laser beam intensity of 10 MW cm^−2^. The stability of UCL emission revealed no photobleaching or photoblinking behavior.

Sequential image acquisition is time consuming and may result in sample drift with image mismatch. One-scan super-resolution FED microscopy achieved super-resolution images simultaneously scanning core-shell Yb^3+^/Tm^3+^,Er^3+^-doped UCNPs with solid and donut excitation light patterns of two light wavelengths, and processing the two-color UCL emission solid and donut images^95^. The Er^3+^-doped NaYF_4_ core produced green UCL emission under excitation with an 808-nm laser beam, while the Yb^3+^/Tm^3+^-doped NaYF_4_ shell emitted blue UCL emission under 940-nm laser beam excitation. To prevent energy transfer between the Yb^3+^/Tm^3+^-doped shell and the Er^3+^-doped core, an inert NaYF_4_ isolation shell was implemented. The lateral resolution was of 54 nm for saturated UCL emission under the solid and donut light pattern excitation with a light intensity of 0.86 MW cm^−2^ and 1.78 MW cm^−2^, respectively. The corresponding super-resolution FED PSF was obtained with a subtraction factor set to 0.95. This approach proves advantageous for prolonged monitoring of live cells because of its drift-free and swift scanning capabilities, the non-bleaching properties exhibited by lanthanide ion-doped UCNPs, and the use of low-intensity NIR CW excitation lasers. Lanthanide ion-doped UCNPs with orthogonal UCL emission spectra under low-intensity light excitation show promise for high-throughput, parallel, multi-channel, low-energy super-resolution applications. This approach can readily integrate with current laser scanning microscopy systems, offering the potential to achieve real-time, low-power super-resolution microscopy through the implementation of instant subtraction in hardware or software.

#### NIRES nanoscopy, NIRB nanoscopy, and the fusion technique

Employing low-photon energy NIR excitation light enhances tissue penetration, reducing autofluorescence and minimizing phototoxicity for live-cell imaging. These principles are applied in fluorescence microscopy using multi-photon excitation (MPE) fluorescence emission, though the longer wavelength compromises resolution^96,97^. While MPE fluorescence microscopy can surpass diffraction limits when combined with techniques like super-resolution STED microscopy, it demands precise alignment and synchronization of high-intensity pulsed lasers, constrained by probe inefficiency. Unlike probes utilized in fluorescence microscopy employing MPE fluorescence emission, which produce fluorescence via simultaneous absorption under high-intensity pulsed laser irradiation, lanthanide ion-doped UCNPs generate UCL emission through a sequential absorption process under low-intensity CW laser irradiation. Consequently, lanthanide ion-doped UCNPs circumvent this limitation, offering significantly lower excitation thresholds compared to the most efficient multiphoton fluorescent probes. Moreover, lanthanide ion-doped UCNPs have the capability to produce UCL emission featuring narrow bandwidth and selectable spectral lines, even within the NIR range.

Taking advantage of these features of lanthanide ion-doped UCNPs, NIRES nanoscopy achieved super-resolution images with negative contrast scanning core Yb^3+^/Tm^3+^-doped UCNPs with a donut excitation light beam at 980 nm and detecting UCL emission at 800 nm^73^. Lanthanide ion-doped UCNPs converted 980-nm excitation photons absorbed by Yb^3+^ ions into 800-nm UCL emission photons originating from the two-photon state ^3^H_4_ of Tm^3+^ ions through an ETU process. In super-resolution imaging of lanthanide ion-doped UCNPs, NIRES nanoscopy used a tightly focused, donut-shaped excitation beam to scan lanthanide ion-doped UCNPs samples, producing negative contrast only when a single lanthanide ion-doped UCNP was precisely centered. Initially limited by diffraction at low excitation laser beam power, NIRES’ optical resolution can surpass this constraint with higher laser beam power through nonlinear excitation (Fig. 3c). The resolution of NIRES nanoscopy for a given excitation light intensity is defined by the intensity saturation curve of lanthanide ion-doped UCNPs. Higher resolution is achieved with a smaller dark center of the donut emission light pattern decreasing the light power value to reach the half value of the maximum *I*_*UCL*_, and the maximum *I*_*UCL*_ for a given light power value at half maximum. Furthermore, higher resolution is achieved with a lower depth of the donut emission light pattern increasing the onset of the curve, that is the value of light power to reach e^−2^ of the maximum *I*_*UCL*_. The lateral resolution was 34 nm for saturated UCL emission under the donut light beam excitation with a light intensity of 4 MW cm^−2^ (Fig. 3d). Moreover, enhancing the resolution is feasible through the optimization of lanthanide ion dopant concentration or by designing a tailored core-shell structure of the lanthanide ion-doped UCNPs. This implies a vast potential for the materials science community to refine the resolution of NIRES nanoscopy.

NIRES-inspired NIRB nanoscopy achieved super-resolution images with negative contrast scanning core Yb^3+^/Tm^3+^-doped UCNPs with a Bessel donut excitation light beam at 980 nm and detecting UCL emission at 800 nm^74^. The use of such a ‘non-diffractive’ light beam and both excitation and emission at the NIR enable high penetration depth with minimal scattering and absorption. The lateral resolution was of 37 nm for saturated UCL emission under the Bessel donut light beam excitation with a light intensity of 10.88 MW cm^−2^. Moreover, NIRES-inspired nanoscopy achieved super-resolution images scanning core Yb^3+^/Tm^3+^-doped UCNPs and using the Fourier domain heterochromatic fusion technique^75^. When excited with a donut light beam at 980 nm with high light intensity, these lanthanide ion-doped UCNPs exhibited UCL emission at 740 nm with a donut light pattern and at 800 nm with a Gaussian-like light pattern because the dark center of the donut was over-saturated. The fusion technique produced super-resolution UCL emission patterns by combining the higher- and low-frequency information from the donut and Gaussian-like UCL emission patterns in the Fourier domain, respectively. The lateral resolution was of 40 nm for saturated UCL emission at 800 nm under the donut light beam excitation with a light intensity of 2.75 MW cm^−2^. Moreover, by harnessing the substantial nonlinear response observed in lanthanide ion-doped UCNPs, as evidenced by recent studies on PA effects^82,83^, the heterochromatic function strategy may significantly enhance imaging resolution.

#### Super-resolution NSIM

SIM uses periodic patterns to down-modulate high spatial frequency information in samples, aiding in the reconstruction of high-frequency details from multiple images acquired with patterned illuminations at varied angles, assisted by optical transfer functions. Recent advancements in denoising methods and modified excitation conditions have led to the development of new variants like NSIM^25,98^, Hessian-SIM^99^, grazing incidence SIM^100^, and multifocal light patterns^101^, known for their enhanced imaging speeds. Super-resolution NSIM achieves a resolution of around 50 nm by utilizing high excitation power to exploit nonlinear saturated photoresponse^25^. However, despite these advancements, deep-penetrating applications face challenges due to sample extinction, resulting in distorted illumination patterns, unwanted out-of-focus light, and reduced emission intensity, compromising both resolution and speed. NIR light MPE techniques, combined with spot scanning, address these challenges but at the expense of slower speed^101^. Organic fluorescent dyes and proteins, commonly used in SIM, require tightly focused, high-power pulsed lasers for MPE due to their small absorption cross-section. However, the high excitation power, particularly in NSIM, constrains the prolonged visualization of subcellular structures in living cells.

The utilization of lanthanide ion-doped UCNPs has facilitated the development of a strategy for super-resolution NSIM, enabling fast super-resolution imaging with deep penetration. Super-resolution NSIM achieved super-resolution images with core Yb^3+^/Tm^3+^-doped UCNPs excited with a sinusoidal light pattern at 976 nm generated by a digital mirror device and detecting UCL emission by a camera^76^ (Fig. 3e). These lanthanide ion-doped UCNPs emitted UCL at 800 nm with high-frequency harmonic peaks in the Fourier transform. When an 80 μm tissue slice was laid atop a layer of lanthanide-ion doped UCNPs, the 800 nm emission pattern effectively reduced scattering while maintaining its pattern, showcasing superior penetration capabilities compared to typical visible UCL emission (Fig. 3f). The latter often suffers from significant distortion and nearly loses structural information. Fourier domain image analysis was used to quantify the information retained in emission patterns using super-resolution NSIM. The use of lanthanide ion-doped UCNPs with both 976-nm excitation and 800-nm UCL emission bands in the NIR range for super-resolution NSIM highlights their superiority over visible emissions, enabling the transmission and detection of structured excitation and emission patterns through thick tissue.

In comparison to the diffraction-limited wide-field imaging case achieved under uniform illumination (*H* = 0) and linear SIM (*H* = 1), the inclusion of additional harmonics *H* in upconversion NSIM (*H* ≥ 2) enhances the lateral resolution to approximately *λ*/[2*NA*(*H* + 1)], where *NA* is the numerical aperture. Adjusting the doping concentrations in lanthanide ion-doped UCNPs allowed for fine-tuning the nonlinearity of the photon response and UCL emission intensity. Lanthanide ion-doped UCNPs with higher Tm^3+^ concentration exhibit stronger harmonic peaks owing to increased nonlinearity derived from enhanced energy transfer from Yb^3+^ to Tm^3+^ and improved CR between Tm^3+^ ions. However, higher doping concentrations often lead to lower emission intensity when using mild excitation power. To achieve an optimized balance between nonlinearity and brightness for imaging quality, a midlevel doping concentration of 4% was selected. Further enhancement in UCL emission intensity was achieved by adjusting the doping concentration of Yb^3+^ ions to 40%, as the optimized Yb^3+^ concentration increased the energy transfer rate from Yb^3+^ to Tm^3+^ ions. Wide-field and super-resolution imaging of the 4% Tm-doped UCNPs was conducted (Fig. 3g). The lateral resolution was 124 nm for saturated UCL emission under the sinusoidal light pattern excitation with a light intensity of 4 kW cm^−2^ (Fig. 3h). In comparison to studies focused on single-point scanning nanoscopy utilizing NIR excitation and NIR UCL emission^73–75^, super-resolution NSIM works without the need for light beam control and achieves higher rates. Furthermore, it uses low excitation light intensity to keep increase of tissue temperature below 3 °C, safe for long-term tracking in living cells^102,103^. Increasing imaging speed with higher excitation power may result in strong photothermal effects. Enhancing the brightness of lanthanide ion-doped UCNPs under low power and applying denoising algorithms like Hessian deconvolution and multifocal patterns^104^ can improve speed and resolution. This method can also enhance imaging depth in various techniques like light sheet-based SIM^105^ and adaptive optics^106,107^.

### Targeted super-resolution techniques based on upconversion inhibition saturation

#### Super-resolution ESA microscopy

**Fig. 4:**
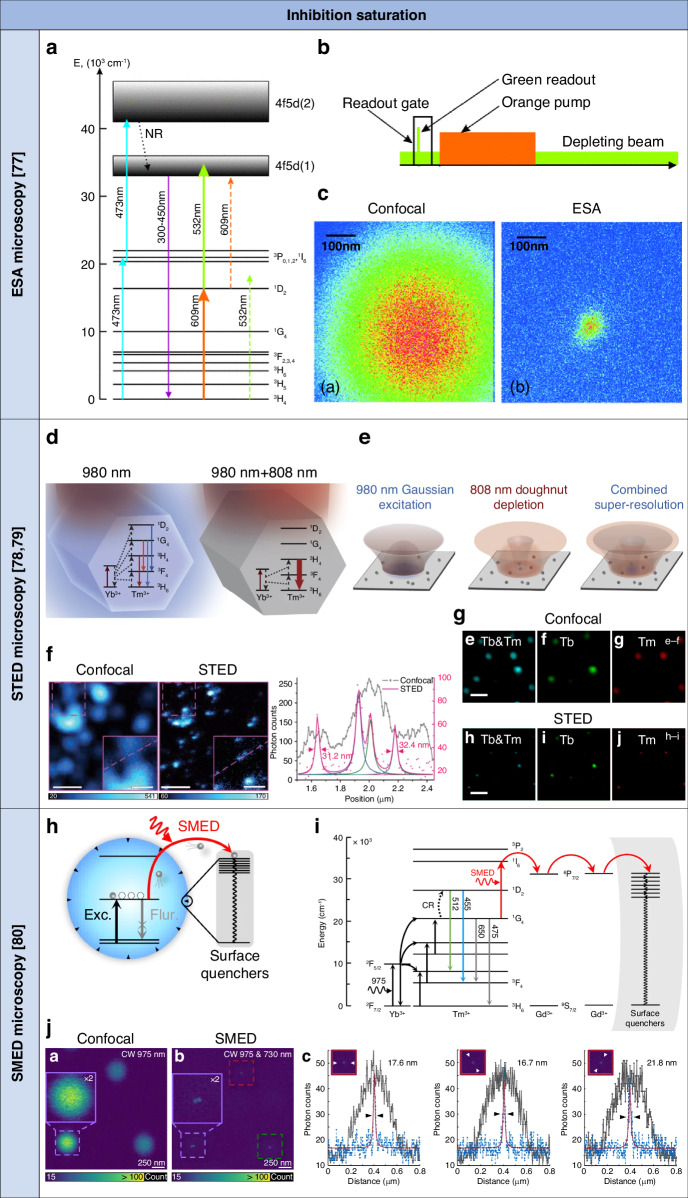
Summary of targeted super-resolution techniques based on upconversion inhibition saturation of lanthanide ion-doped UCNPs. **a** Schematic of the energy level diagram of Pr^3+^-doped UCNPs for super-resolution ESA microscopy. Reproduced with permission from ref. ^77^. Copyright 2011 American Physical Society^77^. **b** Schematic of the sequence of laser pulses for super-resolution ESA microscopy imaging of Pr^3+^-doped UCNPs. Reproduced with permission from ref. ^77^. Copyright 2011 American Physical Society^77^. **c** Confocal and super-resolution ESA microscopy imaging of Pr^3+^-doped UCNPs. Reproduced with permission from ref. ^77^. Copyright 2011 American Physical Society^77^. **d** Schematic of the energy level diagram of lanthanide ion-doped UCNPs with high Tm^3+^ doping for UCL emission excitation and inhibition through STED. Reproduced with permission from ref. ^78^ Copyright 2017 Springer Nature Limited^78^. **e** Schematic of the super-resolution STED microscopy imaging system based on spatially overlapped Gaussian-shaped excitation and donut-shaped depletion laser beams and lanthanide ion-doped UCNPs with high Tm^3+^ doping. Reproduced with permission from ref. ^78^. Copyright 2017 Springer Nature Limited^78^. **f** Confocal and super-resolution STED microscopy imaging, and corresponding intensity profiles of lanthanide ion-doped UCNPs with high Tm^3+^ doping. Scale bars: 500 nm (main images) and 200 nm (insets). Reproduced with permission from ref. ^78^. Copyright 2017 Springer Nature Limited^78^. **g** Dual-color confocal and super-resolution STED microscopy imaging, and corresponding intensity profiles of lanthanide ion-doped UCNPs with high Tm^3+^ doping. Scale bar: 1 μm. Reproduced with permission from ref. ^79^, CC BY 4.0. Copyright 2017 Springer Nature Limited^79^. **h** Schematic of the SMED mechanism for UCL emission depletion through surface migration. Reproduced with permission from ref. ^80^, CC BY 4.0. Copyright 2022 Springer Nature Limited^80^. **i** Schematic of the energy level diagram of lanthanide ion-doped UCNPs with surface quenchers for super-resolution SMED microscopy. Reproduced with permission from ref. ^80^, CC BY 4.0. Copyright 2022 Springer Nature Limited^80^. **j** Confocal and super-resolution SMED microscopy imaging, and corresponding intensity profiles of lanthanide ion-doped UCNPs with surface quenchers. Reproduced with permission from ref. ^80^, CC BY 4.0. Copyright 2022 Springer Nature Limited^80^

Super-resolution ESA microscopy shares similarities with super-resolution STED microscopy in its approach to achieving super-resolution. Both techniques employ a dual-beam setup, with a Gaussian laser beam for excitation and a donut laser beam for inhibition, to selectively activate and deactivate luminescence emission. However, super-resolution ESA microscopy diverges significantly from super-resolution STED microscopy by relying on the principle of stimulated absorption rather than the principle of stimulated emission. Super-resolution ESA microscopy achieved super-resolution images scanning Pr^3+^-doped UCNPs with a time-gated 609-nm Gaussian excitation laser beam, a 532-nm donut inhibition laser beam, and a 532-nm Gaussian excitation laser beam, and detecting UV UCL emission^77^. The UCL emission mechanism involved two sequential excitation steps of Pr^3+^ ions (Fig. 4a)^108^. Firstly, a laser beam at 609 nm triggered the transition of Pr^3+^ ions to their ^1^D_2_ state, characterized by a long lifetime of approximately 150–200 μs. Subsequently, another laser beam at 532 nm facilitated the transition to the emitting 4f5d(1) level, possessing a short lifetime of approximately 18 ns and near-unity UCL emission quantum efficiency. For super-resolution imaging of Pr:YAG nanoparticles, a pulsed 609-nm Gaussian excitation laser beam was used to populate the intermediate excited energy state of lanthanide ion-doped UCNPs, while a CW 532-nm donut inhibition laser beam was used to deplete such a state through ESA except for lanthanide ion-doped UCNPs at the center of the donut. Another pulsed 532-nm Gaussian excitation laser beam excited lanthanide ion-doped UCNPs with a remaining electronic population in the intermediate excited energy state into the emitting excited energy state for UCL emission. Concurrently, photon counts at a photo-detector were selectively gated to include only those photons arriving within a few tens of nanoseconds after the readout pulse, thereby contributing to the readout signal (Fig. 4b). The lateral resolution was 54 nm for saturated upconversion inhibition through ESA to the emitting excited energy state (Fig. 4c). After hours of continuous illumination, there was no change in the UCL emission intensity observed, demonstrating the photostability of Pr:YAG nanoparticles for super-resolution ESA microscopy. Nonetheless, the use of high-photon energy UV UCL emission and a complex excitation scheme, which has limited applicability in biomedical and photonics fields, may prompt the exploration of alternative lanthanide ion-doped UCNPs and illumination conditions for broader applications of this technique. For instance, saturated upconversion inhibition with a depletion ratio of ⁓30% was achieved with Yb^3+^/Er^3+^-doped UCNPs excited at 795 nm and inhibited at 1140 nm with a light intensity at ∼100 kW cm^−2^ through ESA and interionic energy transfer, and detecting green UCL emission^109^. In contrast to the typical super-resolution STED laser beam intensity range of 1–100 MW cm^−2^, this laser beam intensity was up to three orders of magnitude smaller, making it more biologically tissue-friendly.

#### Super-resolution STED microscopy

Super-resolution STED microscopy achieved super-resolution images scanning core Yb^3+^/Tm^3+^ UCNPs with a Gaussian excitation laser beam at 980 nm and a donut depletion laser beam at 808 nm, and detecting UCL emission at 455 nm^78^. The process of photon upconversion was initiated with the absorption of 980 nm excitation by the Yb^3+^ sensitizers, followed by the transfer of this energy onto the scaffold energy levels of the Tm^3+^ emitters (Fig. 4d). Subsequently, UCL emission was generated from the two-photon ^3^H_4_, three-photon ^1^G_4_, or four-photon ^1^D_2_ levels of Tm^3+^. The high doping of 8% of Tm^3+^ ions in these lanthanide ion-doped UCNPs allowed for reduced inter-emitter distance. CR between Tm^3+^ ions excited at 980 nm enabled those higher excited energy states to transfer energy to surrounding Tm^3+^ ions in the ground or lower excited energy states with a PA-like mechanism. This process accelerated the buildup of Tm^3+^ ions in an intermediate excited energy state to establish a population inversion. In the presence of population inversion between the intermediate level ^3^H_4_ and the ground level ^3^H_6_, a laser beam operating at 808 nm, corresponding to the energy gap of the electronic transition from ^3^H_4_ to ^3^H_6_, initiated STED to deplete the ^3^H_4_ level. This process subsequently suppressed UCL emission from higher excited levels. UCL emission at 455 nm from ^1^D_2_ was depleted at 808 nm through STED of the intermediate excited energy state.

A value of light intensity for saturation of inhibition of 0.19 MW cm^−2^ was achieved, which corresponds to about three orders of magnitude reduced light intensity compared with that of typical fluorophores and luminescent nanomaterials such as nitrogen-vacancy centers in diamond and fluorescent quantum dots for super-resolution STED microscopy with a value of light intensity for saturation of inhibition of 1–240 MW cm^−2110–112^. For super-resolution STED microscopy imaging of these lanthanide ion-doped UCNPs, the 808 nm laser beam was spatially modulated to generate a donut-shaped PSF that was spatially overlapped with the Gaussian-shaped PSF of the 980 nm excitation laser beam at the focal plane (Fig. 4e). The lateral resolution was of 28 nm for saturated upconversion inhibition of the intermediate excited energy state to the ground energy state through STED under the 980-nm Gaussian excitation and 808-nm donut depletion light beams with a light intensity of 0.66 MW cm^−2^ and 9.75 MW cm^−2^, respectively (Fig. 4f). Super-resolution STED microscopy images were obtained from the same sample area through continuous laser excitation and scanning, revealing remarkable photostability that persisted for several hours. Super-resolution STED microscopy can be used in different lanthanide ion-doped UCNPs for multicolor super-resolution imaging with the same laser beams. For instance, by adding Tb^3+^ and Eu^3+^ ions to the Yb^3+^-Tm^3+^ system, luminescent nanoprobes with UCL emission in complementary spectral channels can be generated, utilizing energy migration-mediated upconversion beyond the ^1^D_2_ state^113^. Super-resolution STED microscopy achieved two-color super-resolution images scanning core-shell Yb^3+^/Tm^3+^,Tb^3+^ UCNPs with a Gaussian excitation light beam at 975 nm and a donut depletion light beam at 810 nm^79^ (Fig. 4g). UCL emission with two wavelengths was excited at 975 nm and depleted at 810 nm through STED assisted by interionic CR. This demonstration shows promise for multi-color super-resolution STED microscopy, using multiple lanthanide ion emitters, and avoiding coordination of several light beams for excitation and depletion of specific color channels.

Compared to typical super-resolution STED microscopy that allows for real-time investigations at the nanometer scale^114,115^, the limited UCL emission intensity and long UCL emission lifetime of lanthanide ions of UCNPs restricted the scanning speed for super-resolution optical imaging to pixel dwell times of several milliseconds. Fast super-resolution STED microscopy achieved super-resolution images scanning core Yb^3+^/Tm^3+^-doped UCNPs with a Gaussian excitation light beam at 975 nm and a donut depletion light beam at 810 nm, and detecting UCL emission at 455 nm^116^. The high doping of Yb^3+^ ions allowed for enhanced UCL emission through clustering effect^117^, and accelerated kinetics of UCL emission approaching the intrinsic luminescence lifetime of the emitting excited energy states of Tm^3+^ ions. The lateral resolution was of 72 nm. The exposure time was decreased to 10 µs pixel^−1^, which corresponds to about two orders of magnitude reduced exposure time compared to that of previously demonstrated super-resolution STED microscopy using lanthanide ion-doped UCNPs with high Tm^3+^ doping^78,79^. This demonstration shows promise for lanthanide ion-doped UCNP-enabled fast and emission streaking-free super-resolution STED microscopy with an exposure time comparable with that for super-resolution imaging using typical fluorophores and luminescent nanomaterials.

The optimal excitation power for high-efficiency depletion in low-intensity super-resolution STED microscopy using lanthanide ion-doped UCNPs was investigated through rate equation modeling^118^. This method relied on non-saturated excitation based on the dynamic CR energy transfer of lanthanide ion-doped UCNPs. The lateral resolution was 33 nm under a 980-nm Gaussian excitation light beam with a light power of 1 mW and a 808-nm donut depletion light beam with a light intensity of 3.4 MW cm^−2^. Optimizing super-resolution STED microscopy with lanthanide ion-doped UCNPs entails balancing UCL emission, depletion efficiency, and imaging quality, typically through empirical trial and error due to unknown mechanisms. These recent insights into power-dependent nonlinear responses have refined UCL excitation and depletion, enhancing efficiency, and understanding the intricate energy-transfer processes within lanthanide ion-doped UCNPs toward reducing laser beam intensity.

#### Super-resolution SMED microscopy

Lanthanide ion-doped UCNPs, with their long emission lifetimes, have facilitated super-resolution while reducing inhibition laser beam intensity compared to the use of typical counterparts^78,79^. Yet, improving inhibition performance necessitates nanoprobes with larger cross-sections and longer lifetimes, presenting a developmental hurdle. Moreover, prolonged UCL emission lifetimes may lead to slower imaging speeds and weaker UCL emission signals. Therefore, exploring new photophysical mechanisms to enhance UCL emission inhibition while reducing saturation intensity represents a promising avenue. Surface energy quenchers or states frequently impact the UCL emission process in lanthanide ion-doped UCNPs, leading to extensive efforts to alleviate these side effects and enhance UCL emission performance^86^. Conversely, these surface phenomena can stimulate the exploration of innovative mechanisms for optically controlled energy dissipation. Nonetheless, actively controlling surface quenching to deactivate fluorescence has been a considerable challenge. Recently, surface migration was exploited to achieve high-efficiency UCL emission depletion of core gadolinium-doped UCNPs^80^ (Fig. 4h). The gadolinium sublattice established a robust energy migration network, channeling energy from luminescent centers to surface quenchers^113^. This enables the depletion of luminescent center energy via nonradiative dissipation at nanoparticle surfaces, facilitating low depletion saturation intensity. This process initiated with ESA to the ^1^G_4_ state of Tm^3+^, followed by energy pumping to the higher ^1^I_6_ state. Efficient energy transfer then occurred due to the overlap of energy levels between the ^1^I_6_ state of Tm^3+^ ions and the ^6^P_7/2_ state of Gd^3+^ ions, with energy migrating from Tm^3+^ ions to surrounding surface defects or quenchers adjacent to the Gd^3+^ ions (Fig. 4i). Super-resolution SMED microscopy achieved super-resolution images scanning Yb^3+^/Tm^3+^/Gd^3+^-doped UCNPs with a Gaussian excitation light beam at 975 nm and a donut inhibition light beam at 730 nm, and detecting UCL emission at 475 nm (Fig. 4j). UCL emission was depleted with efficiency over 95% and a value of light intensity for saturation of inhibition of 18.3 kW cm^−2^ by surface migration through Gd^3+^ sub-lattices. The lateral resolution was 16.8 nm for saturated upconversion inhibition under the Gaussian excitation and donut inhibition light beams with a light intensity of 98 kW cm^−2^ and 1.09 MW cm^−2^, respectively. The development of this strategy opens up avenues for a more thorough understanding and precise control over nanoparticle surface states, potentially facilitating the further advancement of high-performance luminescent probes for a wide range of low-power, diffraction-unlimited super-resolution imaging techniques and super-resolution photo-activation in applications in biology and photonics, including photo-activation and lithography.

### Targeted super-resolution techniques based on UCL emission non-saturation

#### Super-resolution SEE microscopy

Using luminescent nanomaterials with super-linear emission upon laser illumination enables the confinement of luminescence emission to a smaller region than the laser beam’s width, enhancing imaging resolution. This principle forms the basis for achieving 3D super-resolution on a standard confocal microscope without setup adjustments or complex image processing. However, practical realization was hindered by the lack of availability of suitable nanomaterials with high super-linear properties. Lanthanide ion-doped UCNPs can spontaneously enter a highly super-linear regime, eliminating the need for complex procedures or higher excitation powers. Therefore, lanthanide ion-doped UCNPs have been suggested as a means to demonstrate super-resolution imaging on a confocal microscope^119–122^, but their application was mostly limited to experimentally showcasing lateral resolution enhancement within a proof-of-principle framework.

**Fig. 5:**
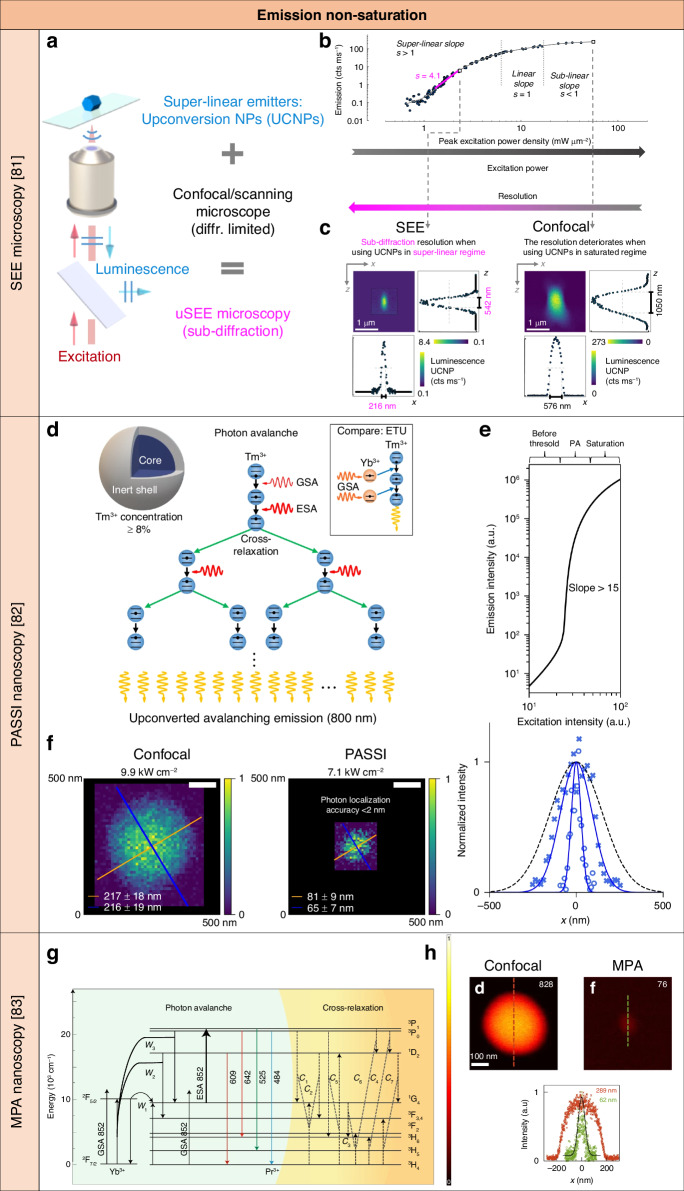
Summary of targeted super-resolution techniques based on UCL emission non-saturation of lanthanide ion-doped UCNPs. **a** Schematic of the experimental setup based on a standard confocal optical microscopy system for super-resolution SEE microscopy using lanthanide ion-doped UCNPs. Reproduced with permission from ref. ^81^, CC BY 4.0. Copyright 2019 Springer Nature Limited^81^. **b** Intensity saturation curve of lanthanide ion-doped UCNPs with high Tm^3+^ doping. Reproduced with permission from ref. ^81^, CC BY 4.0 Copyright 2019 Springer Nature Limited^81^. **c** 3D confocal and super-resolution SEE microscopy imaging, and corresponding intensity profiles of lanthanide ion-doped UCNPs with high Tm^3+^ doping. Reproduced with permission from ref. ^81^, CC BY 4.0. Copyright 2019 Springer Nature Limited^81^. **d** Schematic of the PA effect in Tm^3+^-doped UCNPs. Reproduced with permission from ref. ^82^, 2021. Springer Nature Limited^82^. **e** Model plot of the intensity saturation curve of Tm^3+^-doped UCNPs with PA UCL emission with a non-linearity of more than 15. Reproduced with permission from ref. ^82^. Copyright 2021 Springer Nature Limited^82^. **f** Confocal and PASSI nanoscopy imaging, and corresponding intensity profile of Tm^3+^-doped UCNPs. Reproduced with permission from ref. ^82^ Copyright 2021 Springer Nature Limited^82^. **g** Schematic of the PA mechanism in Yb^3+^/Pr^3+^-doped UCNPs. Reproduced with permission from ref. ^83^. Copyright 2022 Springer Nature Limited^83^. **h** Confocal and MPA nanoscopy imaging, and corresponding intensity profiles of Yb^3+^/Pr^3+^-doped UCNPs. Reproduced with permission from ref. ^83^. Copyright 2022 Springer Nature Limited^83^

Super-resolution SEE microscopy achieved 3D super-resolution images scanning core Yb^3+^/Tm^3+^-doped UCNPs with a Gaussian excitation light beam at 976 nm and detecting UCL emission at 455 nm on a standard confocal optical microscopy system^81^ (Fig. 5a). Such an emission resulted from the ^1^D_2_ → ^3^F_4_ transition of Tm^3+^ ions which involves up to six photons^123^. Typical fluorophores and luminescent nanomaterials require complex conditions and high excitation light intensity to reach a high order of non-linearity^124,125^. On the other hand, the high doping of Tm^3+^ ions of UCNPs allowed for reaching highly super-linear UCL emission spontaneously and with low excitation light intensity. The intensity saturation curve for UCL emission at 455 nm of these lanthanide ion-doped UCNPs showed *n* = 4.1 under non-saturating light excitation at 976 nm (Fig. 5b). A theoretical and computational framework was established to determine the enhancement of optical resolution in both lateral and axial directions when imaging various super-linear fluorophores under diverse excitation beams. Conventional confocal microscopy was utilized when the excitation laser beam intensity fell within the saturation region of lanthanide ion-doped UCNPs’ intensity saturation curve. However, reducing the laser beam intensity to the non-saturation region revealed a highly super-linear dependence of UCL emission, enabling super-resolution microscopy with a confocal microscope in spontaneous super-resolution SEE microscopy mode. The lateral and axial resolutions were 216 nm and 542 nm, respectively, for non-saturated UCL emission under the Gaussian light beam excitation at a light intensity of 2.3 mW μm^−2^ (Fig. 5c). Under super-resolution SEE microscopy imaging conditions, the nanoparticles exhibited high photostability, allowing for hours of imaging without significant deterioration in UCL emission intensity or changes in super-resolution SEE microscopy resolution. Under prolonged high-power illumination, UCL emission from the nanoparticles deteriorated, prompting a change in the intensity saturation curve. To counteract this, a pre-illumination procedure was applied to the nanoparticles, leading to reduced yet stable UCL emission.

In contrast to all previously reported demonstrated super-resolution techniques, super-resolution SEE microscopy using lanthanide ion-doped UCNPs revealed resolution enhancement in the sub-diffraction regime by minimizing the photon budget. This unexpected trend creates opportunities for low-energy super-resolution applications. Although the resolution of super-resolution SEE microscopy using lanthanide ion-doped UCNPs under NIR excitation light is equivalent to that of diffraction-limited techniques using visible excitation light, this technique has advantages such as lower scattering and absorption, photo-damage, and autofluorescence background. Super-resolution SEE microscopy using lanthanide ion-doped UCNPs operates at two to three orders of magnitude lower excitation light intensity compared to that of lanthanide ion-doped UCNP-enabled super-resolution STED microscopy^78,79^. The achievable resolution of super-resolution SEE microscopy may be improved further using lanthanide ion-doped UCNPs with higher order of non-linearity and shorter excitation light wavelength. Also, lanthanide ion-doped UCNPs with shorter luminescence lifetimes and higher brightness, combined with multi-focal approaches, may enable faster imaging.

#### PASSI nanoscopy and MPA nanoscopy

PA is a positive feedback system that leads to an extremely high order of non-linearity and efficiency^42^. Luminescent nanomaterials demonstrating PA behavior with highly nonlinear responses find a compelling application in single-particle super-resolution imaging. This is because the size of the imaging PSF in scanning confocal microscopy decreases inversely with the square root of the degree of nonlinearity. PA-induced non-linearity was first observed in Pr^3+^-doped bulk crystals, which showed a significant rise in UCL emission excited beyond a critical pump light intensity threshold^90^. PA-like behavior was seen in experimental designs using lanthanide ion-doped UCNPs due to aggregation^91^ or pre-avalanche energy looping^42,78,79^ with a high order of non-linearity of up to 6.2 in 8% Tm^3+^-doped UCNPs excited at 976 nm^81^ and up to 3.2 in 1.5% Tm^3+^-doped UCNPs excited at 1064 nm for ESA instead of GSA transitions^126,127^. The concept of using lanthanide ion-doped UCNPs with PA behavior was proposed for super-resolution imaging without photobleaching of fluorophores and simplified optical systems^128^. Theoretical simulations were performed to model PA UCL emission of Nd^3+^-doped UCNPs excited at 1064 nm.

Exploiting the PA behavior of lanthanide ion-doped UCNPs, PASSI nanoscopy achieved super-resolution images scanning core-shell Tm^3+^-doped UCNPs with a Gaussian excitation light beam at 1,064 nm and detecting UCL emission at 800 nm on a standard confocal microscopy system^82^. In these lanthanide ion-doped UCNPs, a single GSA event initiated a chain reaction of ESA and CR events between lanthanide ions, resulting in the UCL emission of many upconverted photons (Fig. 5d). These lanthanide ion-doped UCNPs exhibited the defining features of PA, that are (i) a distinct excitation light intensity threshold at which a significant non-linear rise in population in the excited energy state and UCL emission were detected, (ii) an unusually long rise time, that is the time required to achieve 95% of the asymptotic value, near the PA excitation light intensity threshold, with values reaching a maximum of 608 ms, approximately 400 times the luminescence lifetime of the intermediate excited energy states, iii) ESA that was more than 10,000 times larger than GSA. Importantly, all three PA criteria were satisfied at room temperature for these Tm^3+^-doped UCNPs. Tm^3+^ ions in these lanthanide ion-doped UCNPs absorbed excitation photons at 1,064 nm by non-resonant GSA and resonant ESA, followed by CR and PA UCL emission at 800 nm with an order of non-linearity of more than 15 (Fig. 5e). The resolution in PASSI nanoscopy for a given excitation light intensity was determined by the slope of the intensity saturation curve of these lanthanide ion-doped UCNPs. The lateral resolution was of less than 70 nm for non-saturated UCL emission under the Gaussian light beam excitation at a light intensity of 7 kW cm^−2^ (Fig. 5f). The intrinsic long rise times of more than half a second required to build up the PA effect with these lanthanide ion-doped UCNPs restricted scanning rates. As a result, pixel dwell times of tens to hundreds of milliseconds were required. This limitation may be overcome using multi-point excitation with the potential for scan rates of ⁓4 s or less per frame. These nanoparticles exhibited no measurable photobleaching or hysteresis, indicating an absence of detectable contributions from excitation-induced thermal avalanching^129^.

Recently, a strategy was proposed for propagating the PA effect across different lanthanide ions and core-shell nanostructures of lanthanide ion-doped UCNPs. This approach allowed for realizing high optical nonlinearities from various lanthanide emitters. The key was doping two types of lanthanide ions: PA ions and reservoir ions. PA ions initiated the PA effect, supported by reservoir ions^130^. Yb^3+^ ions were chosen as reservoir ions for their versatility as sensitizers, characterized by a large absorption cross-section and a simple energy-level structure^131^. This selection helped avoid detrimental CR processes while enabling constructive interaction with PA ions. Additionally, the sublattice of Yb^3+^ ions contributed to forming a migration network for energy propagation^132^, activating energy loops that also contributed to the PA effect. Pr^3+^ ions were selected to trigger the PA effect in Yb^3+^ reservoir ions due to their demonstrated PA capability in bulk materials^90,133–136^ and fast UCL emission rate, along with large absorption and UCL emission cross-sections^42^. These Yb^3+^/Pr^3+^-doped UCNPs were excited at 852 nm, and UCL emission generated through the PA effect was detected from Pr^3+^ ions^83^ (Fig. 5g). A CW laser beam at 852 nm, with photon energy matching the ESA in Pr^3+^ ions via the transition ^1^G_4_ → ^3^P_1_, was used for excitation of Yb^3+^/Pr^3+^-doped UCNPs. This wavelength choice resulted in weak non-resonant GSA in both Yb^3+^ and Pr^3+^ ions. Through an efficient energy transfer process, Pr^3+^ ions returned to the ^1^G_4_ state while simultaneously promoting Yb^3+^ ions from the ground state to the excited state. Via an additional energy transfer mechanism, additional Pr^3+^ ions were populated to the ^1^G_4_ state, effectively doubling the population of ^1^G_4_. Subsequently, the strong resonant ESA pumped the Pr^3+^ ions from the ^1^G_4_ to the ^3^P_1_ state, initiating the next looping cycle. This ESA-driven energy transfer formed a positive feedback loop, continuously repeated to facilitate the PA effect. Consequently, it amplified the population of emitting levels, such as the ^3^P_1_ and ^3^P_0_ states, generating UCL emission. For instance, 484-nm UCL emission originated from the transition ^3^P_0_ → ^3^H_4_ and the slope of the intensity saturation curve was up to 26 with a low threshold of about 60 kW cm^−2^.

Super-resolution MPA nanoscopy imaging of Yb^3+^/Pr^3+^-doped UCNPs was performed on a standard confocal microscopy system under excitation from an 852 nm CW laser beam, and detecting 484 nm UCL emission (Fig. 5h). The lateral resolution was of 62 nm for non-saturated UCL emission under the Gaussian light beam excitation at a light intensity of 76 kW cm^−2^, in comparison to a lateral resolution of 289 nm for saturated UCL emission, with the lateral resolution enhanced by approximately fivefold. This demonstration provides a facile route to achieve UCL emissions with an extremely high order of non-linearity and using different lanthanide ion emitters for low-energy super-resolution applications. In contrast to other targeted super-resolution techniques, such as those utilizing lanthanide ion-doped UCNPs through the ESA mechanism with three pulsed or CW laser beams^77,109^, as well as through the STED mechanism with two CW laser beams^78,79^, this technique relied solely on a single NIR CW excitation beam of reduced intensity, eliminating the need for intricate data reconstruction. Also, these lanthanide ion-doped UCNPs demonstrated no photobleaching during hour-long laser scanning imaging, underscoring their remarkable emission efficiency and photostability (Table 1).

**Table 1:**
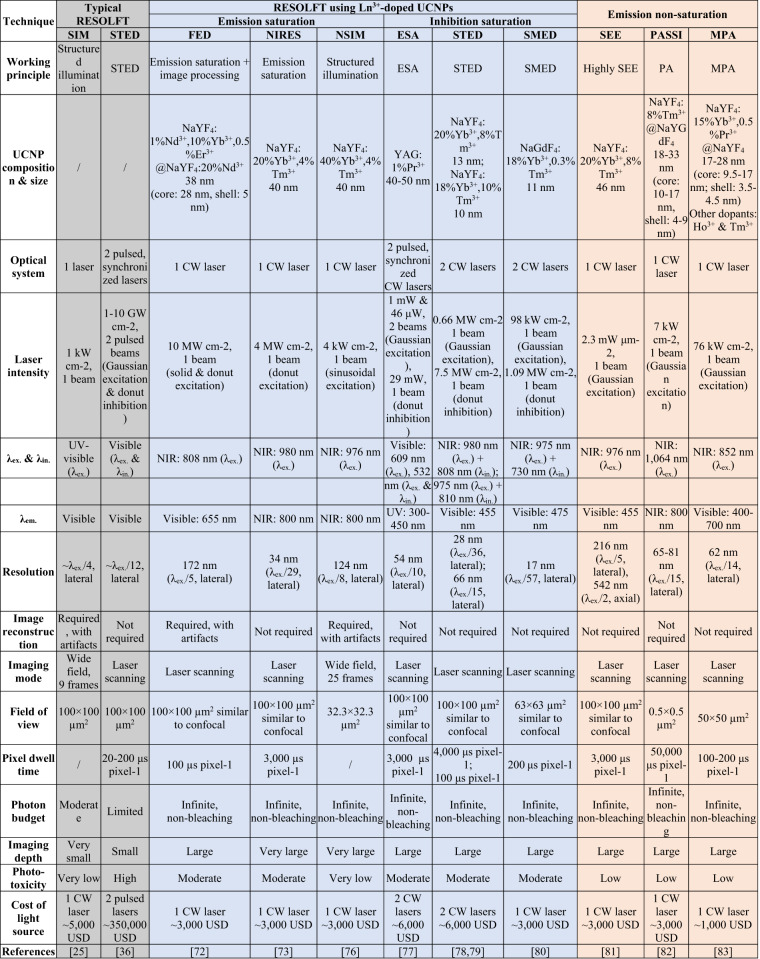
Summary of the working principles and benchmark parameters of typical targeted super-resolution techniques (colored in gray), lanthanide ion-doped UCNP-enabled targeted super-resolution techniques based on upconversion emission saturation and upconversion inhibition saturation (colored in blue), and upconversion emission non-saturation (colored in orange)

## Low-energy super-resolution applications using lanthanide ion-doped UCNPs

### Super-resolution bio-imaging and tracking

Lanthanide ion-doped UCNPs have proven useful in targeted super-resolution techniques for bio-imaging and tracking applications with low energy use^137–139^. This advantage stems from the photon upconversion process of lanthanide ion-doped UCNPs, which requires excitation with low-intensity light and low laser energy density for super-resolution. Lanthanide ion-doped UCNPs were used as luminescent probes to study the morphology and physiology of biological samples, such as subcellular structures and dynamics^140–143^. Lanthanide ion-doped UCNPs outperform typical fluorophores and luminescent nanomaterials used as luminescent probes in various ways. They have a longer luminescence lifetime, higher photostability, and a narrow emission spectrum, which minimizes background noise and increases signal-to-noise ratio. Lanthanide ion-doped UCNPs allow for surface functionalization for improved stability, biocompatibility, and targeting abilities in bio-imaging and tracking applications^144–146^. Lanthanide ion-doped UCNPs can be excited by low-photon energy NIR light, which can penetrate deeper into biological tissues than visible or UV light, making them excellent for in vivo imaging^147^. Single-photon excitation (SPE) techniques require high light power in the visible region to produce high resolution since the tissue attenuates more power for shorter light wavelengths. MPE techniques in the NIR range are frequently used, but the extremely small absorption cross-sections of multi-photon probes compel higher light power. Lanthanide ion-doped UCNPs require minimal light power into the deep tissue, use low-photon energy NIR excitation light wavelength, and have high absorption cross-section. Lanthanide ion-doped UCNPs have enabled to drastically reduce the light intensity needed for saturation of inhibition in targeted super-resolution techniques. Furthermore, when lanthanide ion-doped UCNPs serve as UCL emission imaging probes, they exhibit no background autofluorescence. This is because NIR light does not activate endogenous or exogenous fluorophores in organisms, resulting in a high signal-to-noise ratio. The high photo-stability of lanthanide ion-doped UCNPs allows for their use in both in vitro and in vivo imaging. Their composition of non-toxic elements holds promise for biomedical applications. Moreover, their large, adaptable surface enables straightforward modification for conjugation with hydrophilic ligands, biomolecules, and therapeutic agents, making them well-suited for biological applications.

**Fig. 6:**
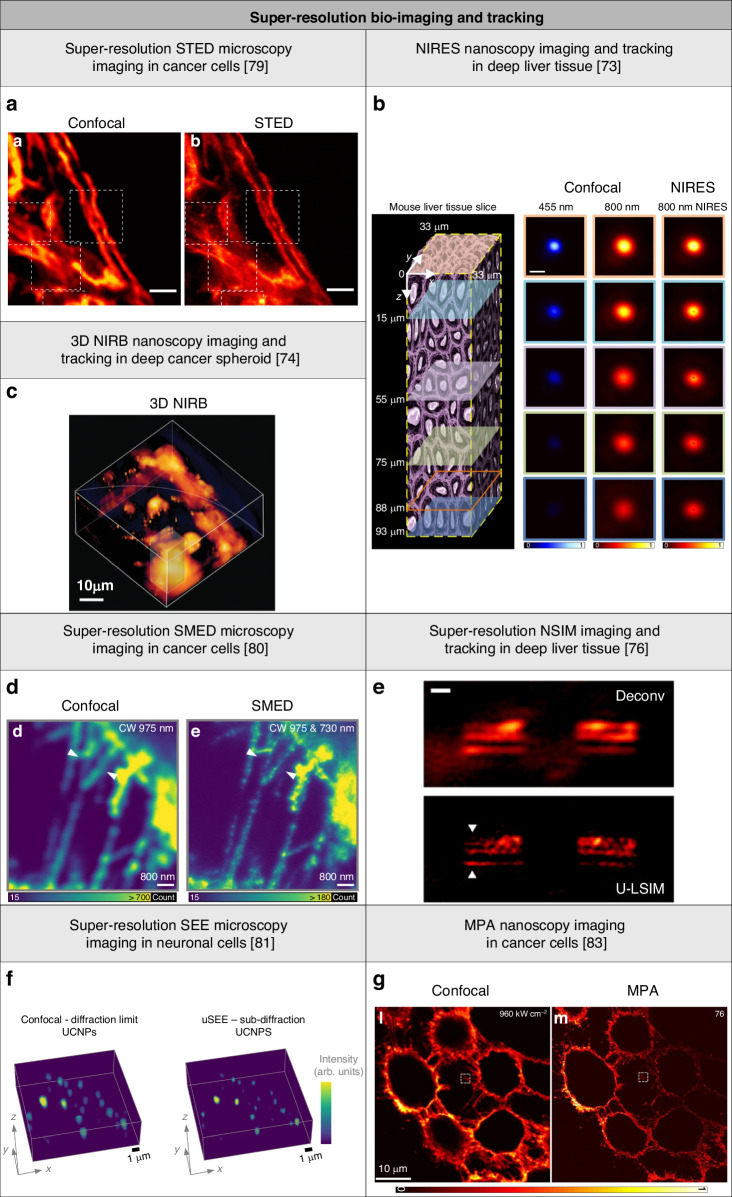
Summary of low-energy super-resolution bio-imaging and tracking applications using lanthanide ion-doped UCNPs. **a** Confocal and super-resolution STED microscopy imaging of the cytoskeleton of HeLa cancer cells using Yb^3+^/Tm^3+^-doped UCNPs. Scale bar: 2 μm. Reproduced with permission from ref. ^79^, CC BY 4.0. Copyright 2017 Springer Nature Limited^79^. **b** Schematic of a mouse liver tissue slice and deep-tissue confocal and NIRES nanoscopy imaging using Yb^3+^/Tm^3+^ UCNPs. Scale bar: 500 nm. Reproduced with permission from ref. ^73^, CC BY 4.0. Copyright 2018 Springer Nature Limited^73^. **c** 3D NIRB nanoscopy imaging of deep cancer spheroid using Yb^3+^/Tm^3+^-doped UCNPs. Reproduced with permission from ref. ^74^. Copyright 2020 John Wiley & Sons, Inc.^74^. **d** Confocal and super-resolution SMED microscopy imaging of the cytoskeleton actin filaments of HeLa cancer cells using Yb^3+^/Tm^3+^-doped UCNPs. Scale bar: 800 nm. Reproduced with permission from ref. ^80^, CC BY 4.0. Copyright 2022 Springer Nature Limited^80^. **e** Wide-field and super-resolution LSIM imaging of deep mouse liver tissue using Yb^3+^/Tm^3+^-doped UCNPs. Scale bar: 1 μm. Reproduced with permission from ref. ^76^. Copyright 2020 American Chemical Society^76^. **f** 3D confocal and super-resolution SEE microscopy imaging of neuronal cells using Yb^3+^/Tm^3+^-doped UCNPs. Reproduced with permission from ref. ^81^, CC BY 4.0, Copyright 2019 Springer Nature Limited^81^. **g** Confocal and MPA nanoscopy imaging of actin protein filaments of HeLa cancer cells using Yb^3+^/Pr^3+^-doped UCNPs. Reproduced with permission from ref. ^83^. Copyright 2022 Springer Nature Limited^83^

Super-resolution STED microscopy imaging of the cytoskeleton of HeLa cancer cells was performed with a lateral resolution of 82 nm using Yb^3+^/Tm^3+^ UCNPs^79^ (Fig. 6a). Deep-tissue NIRES nanoscopy imaging was performed with a lateral resolution of 38 nm using Yb^3+^/Tm^3+^ UCNPs^73^ (Fig. 6b). These lanthanide ion-doped UCNPs were placed behind mouse liver tissue with a thickness of 93 μm and diffused throughout the tissue at various depths. NIRES nanoscopy using NIR light for both excitation and emission enables higher penetration depth, and lower autofluorescence background and phototoxicity in biological samples compared to techniques using visible light. While only 11.3% of UCL emission at 455 nm from these lanthanide ion-doped UCNPs was left in confocal microscopy imaging, 38.7% of UCL emission at 800 nm was detectable in both confocal and NIRES nanoscopy imaging. Lanthanide ion-doped UCNPs were resolved from clusters in deep tissue with a spacing of 72 nm using the NIR-in and NIR-out design. 3D NIRB nanoscopy mapping of a cancer spheroid made of human breast carcinoma MCF-7 cells was performed with Yb^3+^/Tm^3+^-doped UCNPs^74^ (Fig. 6c). These lanthanide ion-doped UCNPs were incubated with the cancer spheroid to enable penetration. The lateral resolution was of 98 nm with a penetration depth of 56 µm. This technique shows promise for studying physiological responses and drug delivery processes by tracking cell uptake and transport of nanoscale cargo in cancer spheroids. Super-resolution SMED microscopy imaging of the cytoskeleton actin filaments of HeLa cancer cells was performed with a lateral resolution of 108 nm using Yb^3+^/Tm^3+^ UCNPs^80^ (Fig. 6d). Deep-tissue LSIM and NSIM imaging was performed with Yb^3+^/Tm^3+^-doped UCNPs^76^ (Fig. 6e). These lanthanide ion-doped UCNPs were placed in nanochannel structures and coated with mouse liver tissue with a thickness of 52 μm. When compared to diffraction-limited wide-field microscopy imaging, super-resolution LSIM and NSIM imaging allowed for improved resolution and signal-to-noise ratio, and an imaging rate of 1 Hz was obtained.

Performing super-resolution bio-imaging and tracking in 3D is desirable, but instrument complexity and large photon budgets limited such an implementation. Only a few targeted super-resolution techniques offered 3D isotropic resolution of less than 30 nm^148–152^. 3D super-resolution SEE microscopy imaging of neuronal cells was performed with a lateral and axial resolutions of 210 nm and 450 nm, respectively, using Yb^3+^/Tm^3+^-doped UCNPs on a standard confocal microscopy system^81^ (Fig. 6f). Continuous super-resolution imaging was performed for over 5 hours with a decrease of UCL emission intensity of less than 1% per hour with potential for super-resolution long-term observations and dynamics studies of biological samples. MPA nanoscopy imaging of sub-cellular actin protein filaments of HeLa cancer cells was performed with a lateral resolution of 62 nm using Yb^3+^/Pr^3+^-doped UCNPs on a standard confocal microscopy system^83^ (Fig. 6g). These lanthanide ion-doped UCNPs enabled imaging speed with an exposure time of 100 μs pixel^−1^, that is approximately 500 times higher than that of the parallel work^82^. Long-term imaging revealed no photobleaching, indicating high UCL emission efficiency and photostability.

Lanthanide ion-doped UCNPs hold potential for super-resolution biophotonics due to their superior photostability and precise detection capabilities^153^. However, concerns about their toxicity arise from their small size, facilitating deep tissue penetration and accumulation in organs, alongside elevated surface reactivity impacting biocompatibility^154–157^. Despite widespread use in biomedicine and generally low toxicity observed, understanding the health impact of engineered nanomaterials is still incomplete, requiring updated regulations^158^. The complexity of nanoparticles complicates toxicity prediction, requiring consideration of various factors like chemical composition, surface properties, and environmental conditions^159–162^. Comprehensive studies on nanoparticle-cell interactions are crucial for accurate toxicity assessment, with further research needed to understand long-term effects and clearance mechanisms for lanthanide ion-doped UCNPs^163^.

#### Super-resolution encoding

Lanthanide ion-doped UCNPs with tunable compositions and structures can be used to encode multiple physical dimensions at the single-nanoparticle level, which can provide low-energy, high-capacity, and high-throughput technologies^164–166^. Lanthanide ion-doped UCNPs are beneficial for use in security and anti-counterfeiting applications because the unique spectral signature of each nanoparticle can be used to encode and detect encrypted information. Luminescence lifetime encoding was achieved using Yb^3+^/Tm^3+^-doped UCNPs by tuning Tm^3+^ ion doping to modulate interionic energy transfer rates and UCL emission lifetimes^167^. UCL emission and luminescence lifetime encoding were achieved using lanthanide ion-doped UCNPs by incorporating energy distributors^168^. Binary temporal upconversion coding was achieved by doping lanthanide ion-doped UCNPs with transition metal Mn^2+^ ions with a long luminescence lifetime of 39 ms and lanthanide ions with a relatively short luminescence lifetime, such as Tm^3+^ ions with a luminescence lifetime of 0.5 ms^169,170^. UCL emission and luminescence lifetime of Mn^2+^ ions in Mn^2+^-doped UCNPs were tuned through crystal-site engineering by alkaline-earth metals doping^171^. Luminescence lifetime and UCL emission wavelength encoding were achieved using lanthanide ion-doped UCNPs by designing multi-shell structures combined with the energy relay technique^172^. Lanthanide ion-doped UCNPs exhibited power-independent orthogonal UCL emission due to the effect of an absorption filtration shell^173^. Lanthanide ion-doped UCNPs exhibited intrinsic time-dependent tunability of the UCL emission wavelength depending on the decay time of the lanthanide ion emitters, using fixed doping and excitation^174^. Luminescence lifetime and UCL emission intensity encoding was achieved using lanthanide ion-doped UCNPs by modulating the sensitizer gradient doping structure^175^. Frequency encoding was achieved using lanthanide ion-doped UCNPs by modulating the phase angle of UCL emission under harmonic-wave excitation^176^.

High spatial-temporal resolution optical multiplexing was used to achieve super-resolution encoding in lanthanide ion-doped UCNPs. Excitation wavelength, emission wavelength, and luminescence lifetime profile encoding were achieved using Tm^3+^-doped and Er^3+^-doped UCNPs^177^. Time-domain optical fingerprints were generated by controlling the populations of the excited energy states in Tm^3+^ and Er^3+^ ions excited at 976 nm or 808 nm. Time-domain confocal microscopy, wide-field microscopy, and super-resolution SIM images were acquired to determine the luminescence lifetime profile of these lanthanide ion-doped UCNPs. The lateral resolution was 184.8 nm under 808-nm pulsed CW light excitation at a light intensity of 5.46 kW cm^−2^. These lanthanide ion-doped UCNPs with high-dimensional optical fingerprints show promise for low-energy super-resolution applications as individually preselectable nanotags. The machine learning (ML) algorithm known as ‘deep learning’ is a helpful tool for rapidly and precisely decoding nanoscale objects^178,179^. Deep learning was used to improve the imaging resolution of lanthanide ion-doped UCNPs with multimodal UCL emissions^180^, decode visible information^181^, and classify the high-dimensional optical fingerprints of lanthanide ion-doped UCNPs based on their luminescence lifetime profiles^182^.

**Fig. 7:**
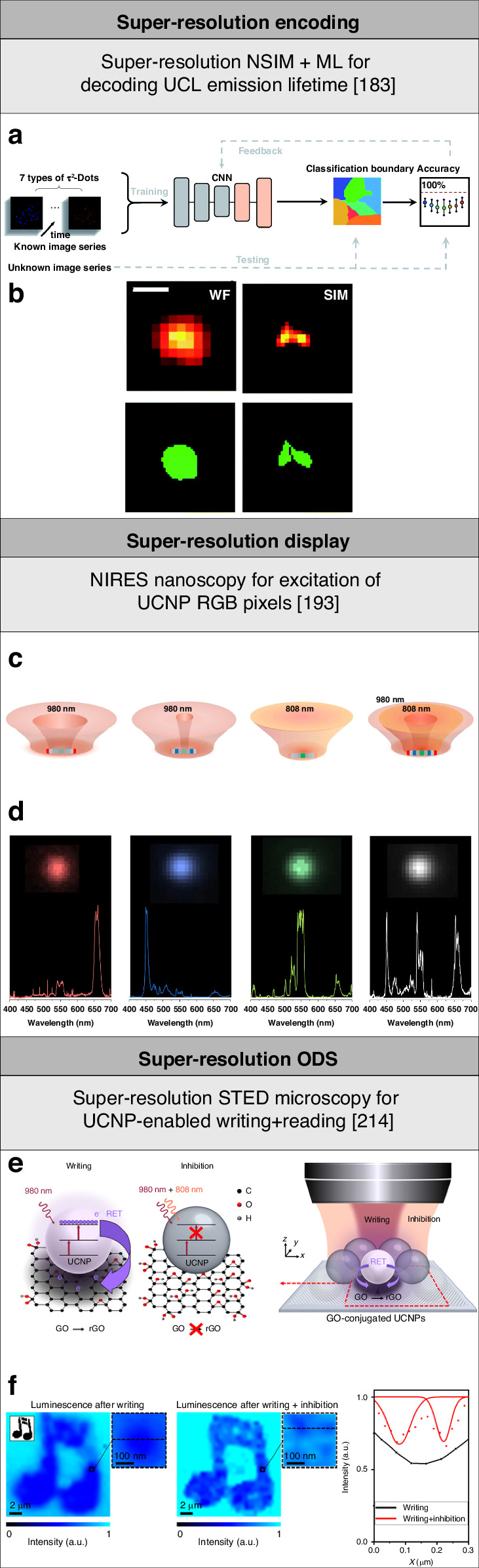
Summary of low-energy super-resolution encoding, display and data storage applications using lanthanide ion-doped UCNPs. **a** Schematic of deep learning assisted super-resolution decoding UCL emission lifetime of lanthanide ion-doped UCNPs. Reproduced with permission from ref. ^183^. Copyright 2022 Royal Society of Chemistry^183^. **b** Time-domain wide-field and super-resolution SIM imaging of lanthanide ion-doped UCNPs decoded using deep learning. Scale bar: 500 nm. Adapted with permission from ref. ^183^. Copyright 2022 Royal Society of Chemistry^183^. **c** Schematic of a nanorod-shaped lanthanide ion-doped UCNP and light excitation patterns for a super-resolution RGB pixel. Reproduced with permission from ref. ^193^, CC BY 4.0. Copyright 2020 Springer Nature Limited^193^. **d** Wide-field microscopy imaging and corresponding UCL emission spectra of a super-resolution pixel emitting RGB and white light using nanorod-shaped lanthanide ion-doped UCNPs. Reproduced with permission from ref. ^193^, CC BY 4.0. Copyright 2020 Springer Nature Limited^193^. **e** Schematic of super-resolution optical data writing using lanthanide ion-doped UCNPs to reduce GO flakes. Reproduced with permission from ref. ^214^, CC BY-NC 4.0. Copyright 2021 American Association for the Advancement of Science^214^. **f** Diffraction-limited and super-resolution optical data readout based on UCL emission quenching of lanthanide ion-doped UCNPs by reduced GO flakes. Reproduced with permission from ref. ^214^, CC BY-NC 4.0. Copyright 2021 American Association for the Advancement of Science^214^

Super-resolution decoding of Nd^3+^, Yb^3+^, and Er^3+^-doped UCNPs with high-dimensional optical fingerprints was achieved using deep learning and time-domain super-resolution LSIM imaging^183^ (Fig. 7a). A deep learning algorithm based on a convolutional neural network architecture used UCL emission lifetime curves of lanthanide ion-doped UCNPs for training to extract distinctive features. After optimization, different types of lanthanide ion-doped UCNPs were randomly selected for testing classification accuracy. The mean classification accuracy for the UCL emission lifetime profiles of these nanoparticles surpassed 90%. Following this, super-resolved decoding was achieved through time-resolved super-resolution LSIM microscopy with deep learning (Fig. 7b). The lateral resolution was 185 nm under 808-nm sinusoidal light pattern excitation with a light intensity of 3.23 kW cm^−2^. The use of super-resolution NSIM^76^ offers the potential for improving decoding resolution compared to the use of super-resolution LSIM. Also, enhancing lanthanide ion-doped UCNPs with shorter lifetimes and brighter emissions could expedite imaging. Further performance enhancements could be achieved by integrating additional features of lanthanide ion-doped UCNPs, such as UCL emission intensity, spectrum, or excitation laser wavelength, into the decoding algorithm.

#### Super-resolution display

Lanthanide ion-doped UCNPs with multi-color UCL emission have the potential to be used in energy-efficient volumetric 3D display technologies as red-green-blue (RGB) emitting pixels^184^. UCL emission from lanthanide ion-doped UCNPs excited using low-photon energy NIR light has a wide spectral range and narrow bandwidth, allowing for precise control of the emission color^185^. Lanthanide ion-doped UCNPs were coated with organic dye-doped nanofilters to achieve pure single-band UCL emissions in the blue at 480 nm, green at 550 nm, and red at 650 nm through radiation reabsorption^186^. These results show promise for bio-detection, optical multiplexing, and RGB emitting pixels for display. The manipulation of the doping and particle sizes of lanthanide ion-doped UCNPs enables to produce UCL emission at specified wavelengths in the red, green, and blue regions of the spectrum. These nanoparticles can thus be used in 3D display technologies as RGB emitting pixels with high resolution, brightness, and energy efficiency. The high stability and strong resistance to photobleaching of lanthanide ion-doped UCNPs are beneficial for their use as RGB emitting pixels in display technologies^187,188^. Lanthanide ion-doped UCNPs can be synthesized with excellent uniformity, which can aid in increasing the stability of the color emitted. The dynamic control of energy transfer between lanthanide ions in UCNPs enabled full-color tunability of UCL emission^189^. These lanthanide ion-doped UCNPs emitted in an extended color gamut through non-steady-state UCL emission by modulating the pulse width of NIR excitation light beams. White light emission was achieved using lanthanide ion-doped UCNPs doped with different lanthanide ions for multiple UCL emissions^190^. The emission colors of these lanthanide ion-doped UCNPs were adjusted by altering the excitation light intensity and modifying the energy transfer pathways between lanthanide ions. Polymer waveguides combined with blue-, green-, and red-emitting lanthanide ion-doped UCNPs excited in the NIR were used to construct flexible transparent displays^191^. An upconversion display system working at a video rate was created based on lanthanide ion-doped UCNPs with improved brightness by optimizing the emitter doping and synchronizing the excitation scanning time to the increased emission time^192^.

Super-resolution pixels emitting RGB and white light were created by synthesizing multi-layer lanthanide ion-doped UCNPs in the shape of nanorods and adjusting excitation light beams^193^. These layers exhibited nonlinear optical responses as well as needed low light intensity for saturation of UCL emission. A lateral resolution of 29 nm was achieved based on NIRES nanoscopy imaging using donut light beam excitation with a light power of 80 mW. These lanthanide ion-doped UCNPs had outer Yb/Er-doped layers that emitted in the red, middle Yb/Tm-doped layers that emitted in the blue, a central Er-doped layer that emitted in the green, and inert NaYF_4_ layers. These layers with bilateral symmetry were selectively activated with specific light excitation wavelengths and patterns, such as donut and Gaussian excitation light beams at 980 nm and 808 nm (Fig. 7c). The red-emitting layers and blue-emitting layers were activated by donut excitation light beams at 980 nm with large and small diameter, respectively (Fig. 7d). The green-emitting layer was activated by a Gaussian excitation light beam at 808 nm. White light emission was produced with these lanthanide ion-doped UCNPs excited simultaneously with a donut light beam at 980 nm and a Gaussian light beam at 808 nm. Furthermore, these lanthanide ion-doped UCNPs enabled super-resolution encoding of emission wavelength by doping different emitters, excitation wavelength by doping different sensitizers, intensity-dependency by doping lanthanide ions with different doping, and luminescence lifetime by modulating interfacial energy transfer across neighboring layers. The controlled synthesis and super-resolution characterizations of anisotropic and heterogeneous lanthanide ion-doped UCNPs provide new scopes for the study of dynamics and directions of energy transfer toward new low-energy super-resolution applications such as displays^194–197^.

#### Super-resolution data storage

Optical data storage (ODS) has become an increasingly important area of research due to the need for sustainable and energy-efficient data storage solutions^198–201^. ODS using lanthanide ion-doped UCNPs has the potential to provide a sustainable and energy-efficient platform for storing large volumes of data. Lanthanide ion-doped UCNPs were used for optical data readout by modulating UCL emission by diarylethene molecules^202^. Optical data writing and erasing were achieved by photoisomerization of diarylethene molecules through radiation reabsorption of orthogonal UV and green UCL emission of lanthanide ion-doped UCNPs excited at 980 nm and 1532 nm, respectively^203^. Lanthanide ion-doped UCNPs with plasmon-enhanced UCL emission were used for fast optical data writing in diarylethene molecules^204^. Diverse geometries of plasmonic nanocraters were produced on metal-insulator-metal combinations and used to modulate UCL emission of lanthanide ion-doped UCNPs for multiplexed ODS and encryption with high storage capacity and throughput^205^. A nanocomposite based on lanthanide ion-doped UCNPs and persistent phosphors were used for orthogonal multiplexed luminescence emission encoding^206^. Lanthanide ion-doped UCNPs with UV UCL emission excited at 980 nm recharged persistent phosphors through energy trapping. The stored energy was then released and transferred to activators, resulting in persistent luminescence. Orthogonal multicolor UCL emission and persistent luminescence were activated in the presence and absence of NIR excitation, respectively. A nanocomposite based on lanthanide ion-doped UCNPs and EuSe semiconductors was used for multi-dimensional ODS leveraging its multiple tunable luminescence^207^.

Super-resolution optical data writing offers a previously unseen approach to high-capacity ODS. However, typical techniques are based on photoinitiation and photoinhibition have high light beam intensity and energy consumption, and limited device lifetime^208–213^. Super-resolution ODS was achieved using Yb^3+^/Tm^3+^-doped UCNPs and graphene oxide (GO) flakes^214^. GO is a graphene-like material^215,216^ that was used for ODS through its reduction to reduced GO^217–219^. A light beam at 980 nm activated the reduction of GO flakes assisted by resonance energy transfer (RET) of quanta with high energy from these lanthanide ion-doped UCNPs (Fig. 7e). A second light beam at 808 nm inhibited the reduction of GO flakes through saturation of upconversion inhibition by preventing the generation of quanta with high energy in these lanthanide ion-doped UCNPs. Super-resolution optical data bits were written with a light intensity of the writing and inhibition beams of 0.13 MW cm^−2^ and 11.25 MW cm^−2^, respectively, and readout by fluorescence quenching microscopy^220,221^ with super-resolution STED microscopy enabled by lanthanide ion-doped UCNPs (Fig. 7f). This strategy enabled to demonstrated a storage capacity of nearly 700 TB per optical disk and potentially achieve an energy consumption of less than 10 µJ bit^−1^. In comparison, Blu-ray Disc technology typically uses picojoules of energy per bit, while optical nanolithography technology typically uses millijoules of energy per bit^222,223^ (Table 2).

**Table 2:** Summary of the working principles and benchmark parameters of low-energy super-resolution applications using targeted super-resolution techniques enabled by lanthanide ion-doped UCNPs

Application	Bio-imaging and tracking							Encoding	Display	Data storage
Technique	STED microscopy	NIRES nanoscopy	NIRB nanoscopy	SMED microscopy	NSIM	SEE microscopy	MPA nanoscopy	NSIM	NIRES nanoscopy	STED microscopy
Operation	Imaging in cancer cells	Imaging and tracking in deep liver tissue	Imaging and tracking in deep cancer spheroid	Imaging in cancer cells	Imaging and tracking in deep liver tissue	Imaging in neuronal cells	Imaging in cancer cells	Decoding UCL emission lifetime, λ_ex._, λ_em._	RGB pixel	Bit writing via UCNP-enabled GO reduction & bit readout via UCL emission quenching
UCNP composition and size	NaGdF_4_:18%Yb^3+^,10%Tm^3+^ (core, 12 nm)	NaYF_4_:40%Yb^3+^,4%Tm^3+^ (core, 41 nm)	NaYF_4_:40%Yb^3+^,4%Tm^3+^ (core, 88 nm)	NaGdF_4_:18%Yb^3+^,0.3%Tm^3+^ (core, 8 nm)	NaYF_4_:40%Yb^3+^,4%Tm^3+^ (core, 40 nm)	NYF_4_:20%Yb^3+^,8%Tm^3+^ (core, 46 nm)	NaYF_4_:15%Yb^3+^,0.5%Pr^3+^ @ NaYF_4_ (core-shell, 26 nm)	Nd^3+^, Yb^3+^, Tm^3+^-doped & Nd^3+^, Yb^3+^, Er^3+^-doped UCNPs(core-shell, 40-60 nm)	NaYF_4_:30%Yb^3+^,2%Er^3+^ (outer layers), NaYF_4_:40%Yb^3+^,4%Tm^3+^ (middle layers), NaYF_4_:15%Er^3+^ (central layer), NaYF_4_ (inert layers) (core-shell, width: 210 nm, diameter: 42 nm)	NaYF_4_:30%Yb^3+^,4%Tm^3+^ (core, 24 nm)
Conjugation/functionalization material	Goat anti-rabbit IgG antibody	Custom-made polymer	Di-block copolymer	Phalloidin molecules	Di-block copolymer	Colominic acid	Phalloidin molecules	/	/	GO flakes
λ_ex._ and intensity/power	975 nm CW (Gaussian)	980 nm CW @ 5.5 MW cm^−2^ (donut)	980 nm CW @ 9.8 MW cm^−2^ (donut Bessel)	975 nm CW @ 98 kW cm^−2^ (Gaussian)	976 nm @ 4 kW cm^−2^ (sinusoidal)	976 nm CW @ 1.7 mW μm^−2^ (Gaussian)	852 nm CW @ 76 kW cm^−2^ (Gaussian)	808 nm @ 5.46 kW cm^−2^ (sinusoidal); 976 nm	980 nm (Gaussian & donut), 808 nm (Gaussian)	980 nm @ 0.13 MW cm^−2^ (Gaussian)
λ_em._	455 nm	800 nm	800 nm	475 nm	800 nm	455 nm	484 nm	Blue, red, NIR (Tm^3+^-doped UCNPs); green, red (Er^3+^-doped UCNPs)	RGB	345 nm, 365 nm, 450 nm
λ_in._ and intensity/power	810 nm CW (donut)	–	–	730 nm CW @ 537 kW cm^−2^ (2.4 mW) (donut)	–	–	/	/	/	808 nm @ 11.25 MW cm^−2^ (donut)
Resolution	82 nm (λ_ex._/12, lateral)	38 nm (λ_ex._/26, lateral)	98 nm (λ_ex._/10, lateral)	108 nm (λ_ex._/9, lateral)	Sub-diffraction	210 nm (λ_ex_/5, lateral), 450 nm (λ_ex_/2, axial)	62 nm (λ_ex_/14, lateral)	184.8 nm (λ_ex_/4, lateral)	29 nm (λ_ex_/34, lateral)	54 nm (λ_ex_/18, lateral)
Exposure time	100 µs pixel^−1^	3000 µs pixel^−1^	3000 µs pixel^−1^	200 μs pixel^−1^	40,000 µs frame^−1^, 1 Hz	3000 µs pixel^−1^	100 μs pixel^−1^	50 μs frame^−1^	3000 µs pixel^−1^	3 × 10^7^ µs bit^−1^ (writing);50,000 µs bit^−1^ (readout)
Reference	^79^	^73^	^74^	^80^	^76^	^81^	^83^	^183^	^193^	^214^

## Research directions and challenges

### Ultrasmall, brighter lanthanide ion-doped UCNPs

Developing lanthanide ion-doped UCNPs with ultrasmall size of less than 10 nm and high brightness is a primary task for their use in low-energy super-resolution applications^224,225^. Lanthanide ion-doped UCNPs for low-energy super-resolution bio-imaging and tracking must be smaller than the subcellular structures being imaged and have a high labeling density. Sub-10 nm lanthanide ion-doped UCNPs are less likely to interfere with the functions of their label target and may even diffuse passively across subcellular structures^226,227^. Super-resolution encoding and RGB pixels for energy-efficient display require ultrasmall and bright lanthanide ion-doped UCNPs with high uniformity and tunability in multiple dimensions at the single-nanoparticle level^228^. Low-energy ODS beyond the diffraction limit aims to reduce the size of optical data bits in order to increase storage capacity while decreasing the overall amount of light used in order to reduce energy consumption. The use of ultrasmall and brighter lanthanide ion-doped UCNPs in low-intensity targeted super-resolution techniques is critical for achieving low-energy super-resolution applications.

**Fig. 8:**
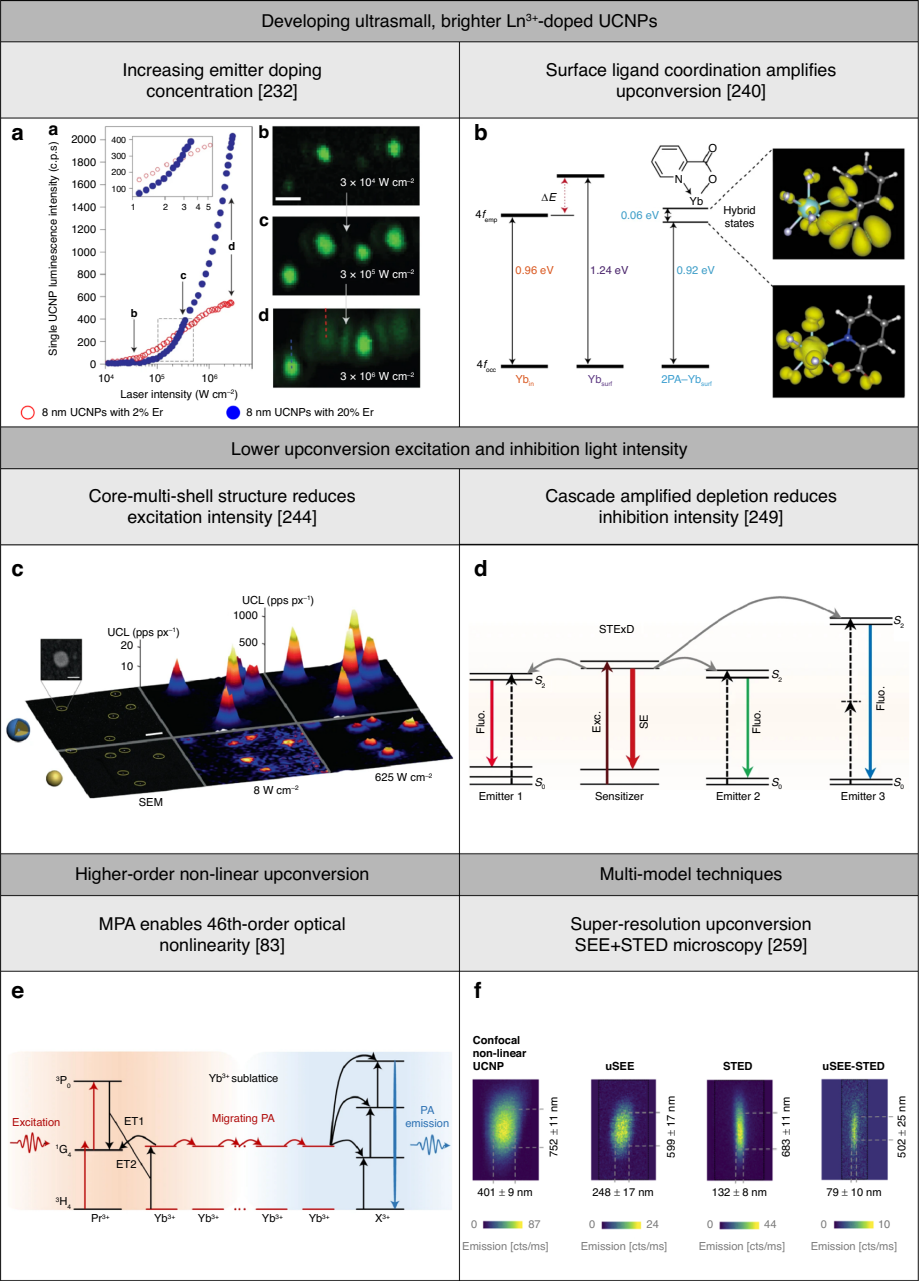
Overview of research directions and challenges of low-energy super-resolution applications using lanthanide ion-doped UCNPs. **a** Ultrasmall lanthanide ion-doped UCNPs with high emitter doping (blue circles) are brighter than nanoparticles with low emitter doping and same size (red circles). Scale bar: 1 μm. Reproduced with permission from ref. ^232^. Copyright 2014 Springer Nature Limited^232^. **b** Ligand coordination induced energy-level reconstruction in ultrasmall lanthanide ion-doped UCNPs for higher UCL emission intensity. Reproduced with permission from ref. ^240^. Copyright 2021 Springer Nature Limited^240^. **c** Lanthanide ion-doped UCNPs with a core-shell-shell structure (top) show higher UCL emission intensity than lanthanide ion-doped UCNPs with a core structure (bottom) under low excitation light intensity of 8 W cm^−2^. Scale bars: 500 nm (main panel) and 30 nm (inset). Reproduced with permission from ref. ^244^. Copyright 2018 Springer Nature Limited^244^. **d** Cascade amplified depletion lowers laser beam intensity for inhibition of UCL emission of lanthanide ion-doped UCNPs. Reproduced with permission from ref. ^249^, CC BY 4.0. Copyright 2022 Springer Nature Limited^249^. **e** The MPA mechanism enables extremely high order of non-linearity of up to 46 in Tm^3+^-doped UCNPs through cascading multiplicative effects of the PA behavior. Reproduced with permission from ref. ^83^. Copyright 2022 Springer Nature Limited^83^. **f** Developing a multi-modal super-resolution technique based on 3D super-resolution SEE microscopy with super-resolution STED microscopy using lanthanide ion-doped UCNPs. Adapted with permission from ref. ^259^. Copyright 2020 Optical Society of America^259^

Compared to typical lanthanide ion-doped UCNPs with size larger than 20 nm, lanthanide ion-doped UCNPs with size smaller than 10 nm are challenging to design and synthesize because their brightness reduces according to the amount of lanthanide ion emitters present. A smaller size enhances the surface area to volume ratio with increased surface-related losses and, as a result, lower brightness^229–231^. Furthermore, the use of high-intensity excitation light to saturate the excited energy states of lanthanide ions that is needed to compensate for surface-mediated energy loss causes photo-damage and high energy consumption. The size- and surface-dependent photophysics of ultrasmall lanthanide ion-doped UCNPs revealed that lanthanide ion emitter doping should be as high as possible without damaging the nanocrystal structure^232^. Lanthanide ion sensitizer doping is less important and may be avoided in single-particle imaging settings. According to these principles, ultrasmall Yb^3+^/Er^3+^-doped UCNPs with high Er^3+^ ion emitter doping were synthesized with sizes of 8 nm and 5 nm. These lanthanide ion-doped UCNPs exhibited UCL emission with more than an order on magnitude higher intensity for single-particle imaging conditions, that is for excitation light intensity of 10^5^–10^7^ W cm^−2^, compared to typical Yb^3+^/Er^3+^-doped UCNPs with low Er^3+^ ion emitter doping and same size (Fig. 8a). Strategies for the synthesis of ultrasmall lanthanide ion-doped UCNPs with higher brightness and optical stability include the optimization of the host matrix and lanthanide ion doping, and coating lanthanide ion-doped UCNPs with inert shells^233,234^. Despite its efficacy, passivating lanthanide ion-doped UCNPs with an optically inactive shell enlarges particle size. Alternative methods, like high-power excitation to saturate lanthanide ion excited states, intensify UCL emission but risk tissue damage and require complex instrumentation.

Investigating complex optical pathways, including energy migration, CR, and vibronic dissipation, is crucial for addressing the challenge of achieving high brightness in ultrasmall lanthanide ion-doped UCNPs. Recent studies reported on the use of organic molecules to control UCL emission of lanthanide ion-doped UCNPs^235–237^. Interactions between lanthanide ion-doped UCNPs and organic molecules involving energy transfer between lanthanide ions and molecular triplet states enabled the generation of visible UCL emission under NIR excitation^238^. Furthermore, UCL emission from different lanthanide ion emitters was achieved through the energy relay technique mediated by molecular triplet excitons^239^. Ultrasmall Yb^3+^/Tm^3+^-doped UCNPs were conjugated with bidentate picolinic acid molecules to achieve strong UCL emission^240^. Ligand-coordinated ultrasmall lanthanide ion-doped UCNPs showed a 11,000-fold enhancement of four-photon UCL emission excited at 980 nm (Fig. 8b). Mechanistic studies revealed that surface ligand coordination modified orbital properties, reducing the energy gap between surface and 4*f* orbitals of inner lanthanide sensitizers. This adjustment facilitated energy migration within the ytterbium sublattice, minimizing energy loss to surface defects and enhancing energy transfer to the emitters. Additionally, ligand coordination induced energy-level reconstruction, maintaining separations exceeding 2 nm between ligands and sensitizers. These findings showcased a new pathway for developing highly luminescent ultrasmall lanthanide ion-doped UCNPs and UCNP-based nanohybrids and manipulating surface states toward low-energy super-resolution applications.

#### Lower upconversion excitation and inhibition light intensity

Achieving strong UCL emission under low-intensity light excitation is critical for low-energy super-resolution applications. However, the 4f-4f electronic configurations of lanthanide ions make UCL emission of lanthanide ion-doped UCNPs a low-efficiency process. In addition, unfavorable interactions with surface quenchers reduce the UCL emission intensity. Excitation light intensities of ⁓10^5^–10^7^ W cm^−2^ and prolonged irradiation are typically used for single-particle imaging, resulting in potential photo-damage and high energy consumption. Lanthanide ion-doped UCNP design and synthesis have advanced to boost UCL emission using low excitation light intensity^241–243^. UCL emission of lanthanide ion-doped UCNPs was studied at the single-particle level using wide-field and confocal microscopy imaging across a wide range in excitation light intensity, spanning from 6 MW cm^−2^ to 8 W cm^−2^^244^ (Fig. 8c). The dependence on the size, the presence of an inert shell, and changes in lanthanide ion emitter doping of lanthanide ion-doped UCNPs were studied to maximize their brightness. A new formulation of core-shell-shell NaYF_4_@NaYb_1-x_F_4_:Er_x_@NaYF_4_ UCNPs with a high Yb^3+^ ion doping was demonstrated to be brighter than typical core NaY_0.78_F_4_:Yb_0.2_Er_0.02_ UCNPs. Due to their high Yb^3+^ ion sensitizer doping and inert shell to avoid energy migration to defects, these 8%Er^3+^ core-shell-shell UCNPs showed a 150-fold increase of UCL emission at 8 W cm^−2^. Alternatively, UCL emission of lanthanide ion-doped UCNPs was boosted through the strategic manipulation of the local environment. The excitation light intensity in nonlinear saturation super-resolution microscopy was reduced by mounting lanthanide ion-doped UCNPs atop a mirror to shift the saturation light intensity threshold of UCL emission to the lower excitation light intensity pumping area^245^. The interference in the local electromagnetic field limited the PSF to a specific spot^246^, increasing excitation efficiency and enabling UCL emission saturation at a 10-fold lower excitation light intensity. A lateral resolution of about 35 nm was obtained using donut light beam excitation at 980 nm with a light intensity of 1.6 MW cm^−2^, needing a 3-fold lower saturated excitation light intensity compared to that needed with non-mirror imaging settings.

In order to achieve super-resolution through saturated upconversion inhibition of targeted super-resolution techniques, the inhibition light intensity is raised above the excitation light intensity, resulting in a high light beam intensity with potential photo-damage and a high energy consumption. Alternative mechanisms for upconversion inhibition must be investigated by researching new compositions and structures of lanthanide ion-doped UCNPs, routes to control UCL emission under excitation and inhibition light, and excitation and inhibition light wavelengths. STED revealed distinct processes for upconversion of Yb^3+^/Er^3+^-doped UCNPs^247^. Lanthanide ion-doped UCNPs demonstrated that STED of the intermediate excited energy state modulated UCL emission at 655 nm excited at 980 nm and depleted at 1540 nm. In contrast, it had less of an effect on UCL emission at 525 nm and 545 nm. The differences in behavior of green and red UCL emissions from these lanthanide ion-doped UCNPs were linked to their distinct photophysical processes. STED of UCL emission of Yb^3+^/Tm^3+^-doped UCNPs was reported using excitation light at 980 nm and depletion light at 1550 nm with both light intensities of less than 100 W cm^−2^^248^. The discovery of stimulated-emission induced excitation depletion (STExD) unveiled a low-power mechanism to suppress UCL emission of multi-chromatic lanthanide ion-doped UCNPs using low-power NIR CW laser beams^249^ (Fig. 8d). Cascade amplified depletion in Nd^3+^-doped UCNPs inhibited UCL emission across various lanthanide ion emitters (e.g., Nd^3+^, Yb^3+^, Er^3+^, Ho^3+^, Pr^3+^, Eu^3+^, Tm^3+^, Gd^3+^, and Tb^3+^). To achieve this, Nd^3+^ was employed as the sensitizer^250^, leveraging its typical quasi-four-level configuration favorable for lasing^251^. Notably, the long-lived ^4^F_3/2_ state of Nd^3+^ contrasts with the rapidly decaying ^4^I_11/2_ state, enabling a large emission cross-section and the establishment of population inversion between these states. As a result, the depletion saturation intensity was notably lower, even by one or two orders of magnitude compared to organic dyes. Nd^3+^-doped UCNPs was used to achieve an ultrahigh depletion efficiency of 99.3 ± 0.3% for the 450 nm UCL emission under excitation at 740 nm and depletion at 1064 nm, accompanied by a low saturation intensity of 23.8 ± 0.4 kW cm^−2^. Furthermore, super-resolution STExD microscopy bio-imaging was demonstrated with a lateral resolution of 34 nm using a 740-nm Gaussian-shaped excitation beam at 78.3 kW cm^−2^ and a 1064-nm donut-shaped depletion beam at 4.5 MW cm^−2^. The ability of lanthanide ion-doped UCNPs to inhibit upconversion using low-intensity light is a desirable property. Ongoing research in this area will be critical for advancing the development of optimized and new mechanisms for upconversion inhibition capable of providing high-resolution and sensitivity methods while consuming less energy and lowering the possibility of photobleaching and photodamage.

#### Higher-order non-linear upconversion

The ability to generate large optical non-linearity from single lanthanide ion-doped UCNPs is critical for many low-energy super-resolution applications. The super-linearity of UCL emission was optimized by adjusting lanthanide ion-doped UCNP composition and structure to improve resolution and reduce excitation light intensity^252^. Lanthanide ion-doped UCNPs with a high Yb^3+^ ion sensitizer doping were studied at various lanthanide ion doping ratios and throughout a wide range of excitation light powers. These lanthanide ion-doped UCNPs achieved a maximum slope value of 7.2 in the intensity saturation curve. Super-resolution SEE microscopy images were acquired by scanning these lanthanide ion-doped UCNPs using a Gaussian excitation light beam at 976 nm. The lateral resolution showed a 2-fold improvement compared to the diffraction limit for a light intensity of 46 kW cm^−2^. Super-resolution STED microscopy images were acquired by scanning these lanthanide ion-doped UCNPs with a Gaussian excitation light beam at 976 nm and a donut depletion light beam at 808 nm. The lateral resolution was of 60 nm for excitation and depletion light intensity of 49 kW cm^−2^ and 4.1 MW cm^−2^, respectively. The excitation light beam intensity for super-resolution STED microscopy using these lanthanide ion-doped UCNPs with higher order non-linear UCL emission was reduced to values similar to those used in super-resolution SEE microscopy. The control of these lanthanide ion-doped UCNPs’ intensity saturation curves enabled optical multiplexing capabilities with the potential for super-resolution imaging and decoding distinct lanthanide ion-doped UCNPs in a mixture excited at different light beam intensities.

UCL emission with extremely high order of non-linearity was achieved through the MPA mechanism using lanthanide ion-doped UCNPs doped with various lanthanide ion emitters^83^ (Fig. 8e). Lanthanide ion-doped UCNPs were synthesized with a core made of Yb^3+^ and Pr^3+^ ions that activated avalanche looping cycles. An energy migration network based on Yb^3+^ ions with high doping^253^ propagated the non-linear response from the core to the shells that were doped with different lanthanide ion emitters such as Ho^3+^ and Tm^3+^ ions. This event resulted in even higher order non-linearity through cascading multiplicative effects. The nonlinearity of 452-nm UCL emission (^1^D_2_ → ^3^F_4_) corresponding to the four-photon process of Tm^3+^ ions was multiplied by that of Pr^3+^ ions through the MPA process and an extremely large slope of up to 46 with a threshold of 120 kW cm^−2^ was observed in the intensity saturation curve for these lanthanide ion-doped UCNPs. The 852-nm CW excitation laser beam could not generate UCL emission by directly exciting the Yb^3+^-Tm^3+^ system, confirming that the observed UCL emission from Tm^3+^ ions in MPA nanoparticles originated from an energy transfer from the Yb^3+^/Pr^3+^-doped core. The amplification of the order of non-linearity enabled by the MPA mechanism and the use of diverse lanthanide ion emitters show promise for low-energy 3D super-resolution applications using lanthanide ion-doped UCNPs doped with different lanthanide ions. This strategy may enable to produce highly non-linear optical responses in lanthanide ion-doped UCNPs with tunable size, doping, structure, high brightness and multiple UCL emissions^254–256^.

#### Multi-modal super-resolution techniques using lanthanide ion-doped UCNPs

Achieving multi-modality through the combination of various targeted super-resolution techniques enables the simultaneous visualization of various sample components, providing complementary information and enhancing the overall performance^257,258^ The integration of multiple targeted super-resolution techniques using lanthanide ion-doped UCNPs may provide combined benefits for low-energy super-resolution applications. Because super-resolution STED microscopy and 3D super-resolution SEE microscopy using lanthanide ion-doped UCNPs are complimentary techniques, combining them has the potential to provide benefits in terms of maximum achievable 3D resolution as well as reducing the excitation and inhibition light beam intensity. Super-resolution SEE microscopy was combined with super-resolution STED microscopy using Yb^3+^/Tm^3+^-doped UCNPs^259^ (Fig. 8f). Both super-resolution techniques relied on lanthanide ion-doped UCNPs that exhibited UCL emission at 455 nm excited at 976 nm. 3D super-resolution SEE-STED microscopy images were acquired by scanning these lanthanide ion-doped UCNPs with a Gaussian excitation light beam at 976 nm and a donut depletion light beam at 808 nm. This multi-modal technique enabled a lateral resolution of 79 nm due to super-resolution STED microscopy and an axial resolution of 502 nm due to super-resolution SEE microscopy. The excitation light intensity was 2 µm µm^−2^, corresponding to the super-linear regime for these lanthanide ion-doped UCNPs, and the depletion light intensity was of 94 mW µm^−2^. Researchers and engineers may engage in optimizing the morphological and optical properties of lanthanide ion-doped UCNPs for combined targeted super-resolution techniques. This involves exploring tailored optical systems and employing computational methods to synergize expertise across disciplines like materials science, chemistry, optics, and computational modeling with the aim of improving resolution and reducing excitation and inhibition light beam intensities.

## Conclusion and outlook

A substantial amount of research has focused on the development of targeted super-resolution techniques that control optical transitions of lanthanide ion-doped UCNPs using low-intensity excitation and inhibition light. These techniques have allowed for achieving low-energy super-resolution applications with a broad impact in the life sciences and other field of technology. We described the dynamics of lanthanide ion-doped UCNPs for super-resolution under low-intensity light and their use in targeted super-resolution techniques. We highlighted low-energy super-resolution applications of lanthanide ion-doped UCNPs, including bio-imaging and tracking, encoding, display, and data storage, as well as the related research directions and challenges in the field. Lanthanide ion-doped UCNPs have demonstrated beneficial as luminescent and photo-activation nanomaterials in low-energy super-resolution applications with high resolution, photostability, tunability, and penetration depth, as well as low-intensity excitation and inhibition light with low energy consumption.

Despite significant research efforts by the materials science and optics communities, there are a number of challenges with lanthanide ion-doped UCNPs for low-energy super-resolution applications. The low UCL emission quantum yield observed in lanthanide ion-doped UCNPs has necessitated the development of new pathways for improving UCL emission intensity by engineering lanthanide ion doping and UCNP structure^260^, increasing excitation light intensity^261^, combating surface quenching^262,263^, using plasmonic enhancement^264–267^, and using heat stimulation^268^. Extending the restricted number of excitation, inhibition, and emission wavelengths of lanthanide ion-doped UCNPs due to the 4*f* electronic configurations of lanthanide ions may allow for the manipulation of UCL emission for specific applications and settings^269^. Lanthanide ion-doped UCNPs with tailored properties may advance with the use of new smart tools and methods such as ML algorithms^270–272^, resulting in the development of more effective and efficient lanthanide ion-doped UCNPs for use in low-energy super-resolution applications in the life sciences and other technological fields.

Broadening the scope of tuning UCL emission of lanthanide ion-doped through low-intensity light irradiation and in conjunction with diverse stimuli presents numerous possibilities for enhancing the application of these nanoparticles in a range of cutting-edge, low-energy super-resolution applications. Several techniques have been developed to remotely tune UCL emission of lanthanide ion-doped UCNPs^273^. These methods include the modulation of light irradiation^274^, ion beam irradiation^275^, and the electric^276,277^ and magnetic fields^278,279^. These features of lanthanide ion-doped UCNPs have the potential to allow for expanding their use in new frontier super-resolution applications in bioimaging, diagnostics, therapeutics, environmental monitoring, optoelectronics, information technology. and beyond, which require low energy consumption^280–282^. Furthermore, exploring further avenues for new low-energy super-resolution applications using lanthanide ion-doped UCNPs involves leveraging their adaptability in combination with other nanomaterials to create nanocomposites and nanohybrids^283^. Lanthanide ion-doped UCNPs can be readily incorporated into host matrices, deposited into films, conjugated with other nanomaterials to form nanocomposites, surface functionalized, and integrated with micro-/nano-structures. Numerous strategies have been developed to incorporate lanthanide ion-doped UCNPs and other functional materials into a cohesive nanosystem^284–287^. Current research extensively delves into the construction strategies and synthesis methods for nanocomposites based on lanthanide ion-doped UCNPs, primarily organized into three categories: self-assembly, in-situ growth, and epitaxial growth^288^.

A challenge in utilizing lanthanide ion-doped UCNPs for low-energy super-resolution applications in life sciences and other technological fields is ensuring scalability and reproducibility in their synthesis. Transitioning in-lab lanthanide ion-doped UCNPs to scalable production requires addressing cost, material compatibility, and scalability^289^. Synthesis methods for these UCNPs are typically divided into batch reactors^290–292^, with pot-by-pot production, and continuous reactors^293–295^, featuring uninterrupted kinetic processing and continuous flow. Both face challenges in reproducibility and yield, with a major obstacle being the substantial solvent requirement, leading to uneven precursor concentrations and temperature distributions. This disrupts thermodynamic equilibrium, impacting particle properties and luminescent performance^296,297^. Overcoming these hurdles is vital to fully harness the potential of lanthanide ion-doped UCNPs for wider low-energy super-resolution applications and commercial use. The continuous preparation method shows promise, offering benefits like improved mixing and cost reduction. However, for effective industrial production of UCNPs, parameters like throughput and yield need further investigation^298,299^. Better understanding of mixing mechanisms and advanced correlations between nanoparticle properties are also essential for future scale-up and mass production.

**Fig. 9:**
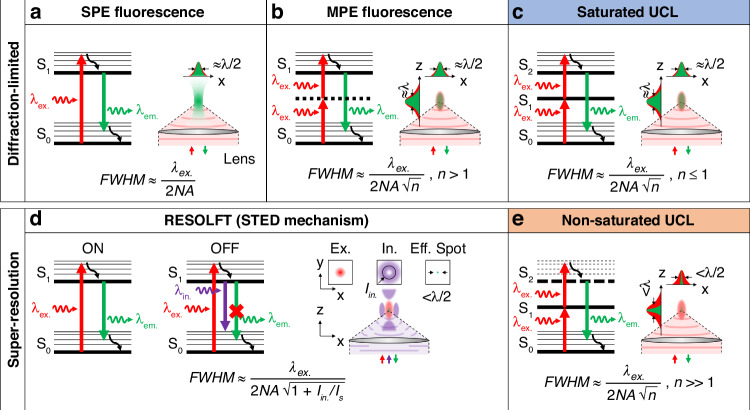
Overview of the working principles of diffraction-limited (standard) and targeted super-resolution techniques based on fluorescence and UCL emission. **a** Schematic of SPE fluorescence emission of a fluorophore through absorption of one photon at *λ*_*ex*._ and emission at *λ*_*em*._, and resolution of fluorescence microscopy based on SPE fluorescence emission. **b** Schematic of MPE fluorescence emission of a fluorophore through simultaneous absorption of multiple photons mediated by a virtual state and resolution of fluorescence microscopy based on MPE fluorescence emission. **c** Schematic of UCL emission of lanthanide ion-doped UCNPs through sequential absorption of multiple photons mediated by a real state and resolution of UCL microscopy based on saturated UCL emission. **d** Schematic of RESOLFT based on the STED mechanism through saturation of bright (ON) and dark (OFF) states under irradiation at *λ*_*ex*._ and *λ*_*in*._, respectively, and resolution of RESOLFT techniques. **e** Schematic of UCL emission of lanthanide ion-doped UCNPs and resolution of UCL microscopy based on non-saturated UCL emission. Real and saturated states are represented by solid lines, while virtual and non-saturated states are represented by dotted and dashed lines, respectively. *S*_*0*_=ground energy state; *S*_*1*_=excited energy state; *S*_*2*_=higher-lying excited energy state; *λ*_*ex*._=excitation wavelength; *λ*_*em*._=emission wavelength; *λ*_*in*._=inhibition wavelength

Box 1 Fundamentals of lens-based optical microscopy Lens-based optical microscopy uses a lens to focus light onto a sample and then collect the light that the material emits, transmits, or reflects to generate an image. Fluorescence microscopy is a widely used technique which produces optical images by detecting fluorescence emission, which is light emission by a medium called a ‘fluorophore’ that has absorbed light with a shorter excitation wavelength *λ*_*ex*._ than the emission wavelength *λ*_*em*._. For fluorescence microscopy based on single-photon excitation (SPE) fluorescence emission—the most popular lens-based optical microscopy technique—the lateral resolution is typically measured by the full width at half maximum (FWHM) of the focal spot, that is ⁓*λ*_*ex*._/2*NA*, where *NA* is the numerical aperture of the objective (Fig. 9a)^300–303^. Different from SPE, multi-photon excitation (MPE) is based on the simultaneous absorption of multiple photons mediated by a virtual state to excite fluorophores from one energy state to a higher one^304–307^. For fluorescence microscopy based on MPE fluorescence emission, the lateral resolution is ⁓*λ*_*ex*._/2*NAn*^1/2^, where *n* is the excitation photon number (Fig. 9b). This technique provides 3D resolution because the rate of MPE depends non-linearly on the excitation power *P*, that is *I*_*fl*_∝*P*^*n*^, where *I*_*fl*_ is the fluorescence emission intensity^308,309^. In practice, MPE has a restricted order of non-linearity of *n* < 4 and requires a longer *λ*_*ex*_. than SPE, offsetting any resolution enhancement owing to non-linearity.

Another technique is UCL microscopy, which uses UCL emission from luminescent nanomaterials such as lanthanide ion-doped UCNPs. Different from MPE fluorescence emission, UCL emission is based on the sequential absorption of multiple photons mediated by a real state to excite lanthanide ions of UCNPs from one energy state to a higher one. UCL emission of lanthanide ions of UCNPs is a super-linear process with an order of non-linearity of *n* > 1 (typically *n* = 2 or 3) for *P* < *P*_*s*_, where *P*_*s*_ is the excitation power to saturate optical transitions of lanthanide ions of UCNPs, while it becomes linear with *n* = 1 and sub-linear with *n* < 1 for *P* = *P*_*s*_ and *P* > *P*_*s*_, respectively (Fig. 9c). However, standard optical microscopy is limited by light diffraction, which results in a resolution limit of about half the light wavelength used to image the sample.

Far-field super-resolution microscopy has overcome this limitation through using a variety of techniques, including deterministic (also known as ‘targeted’) super-resolution techniques, that minimize the size of the point spread function (PSF), and stochastic super-resolution techniques, that precisely localize individual fluorophores^310–315^. The concept of ‘reversible saturable optical (fluorescence) transition’ (RESOLFT)^316–319^ was established to encompass targeted super-resolution techniques that achieve super-resolution by exciting a fluorescent molecule to a bright ‘ON’ state with an activation laser and then de-exciting the molecule to a dark ‘OFF’ state with a second inhibition laser. By selectively saturating the fluorescence emission of molecules in the periphery of the excitation spot, a smaller effective excitation spot is generated, leading to higher resolution. For RESOLFT techniques such as super-resolution stimulated emission depletion (STED) microscopy^320^, the lateral resolution is ⁓*λ*/2*NA*(1*+I*_*in*._*/I*_*s*_)^1/2^, where *I*_*in*._ is the light intensity at the spatial gradient maximum at the inhibition wavelength *λ*_*in*._, and *I*_*s*_ called ‘saturation intensity’ is the light intensity that outperforms the competing optical transition (Fig. 9d).

A different method to achieve super-resolution is based on luminescent nanomaterials with a high order of non-linearity of n»1. These nanomaterials emit significantly only at the maximum of the spatial gradient, i.e. from a region smaller than the size of the focal excitation light intensity pattern itself, resulting in increased resolution. Progress in materials science and optics has enabled to develop lanthanide ion-doped UCNPs exhibiting UCL emission through new upconversion mechanisms, such as highly super-linear upconversion and photon-avalanche (PA) upconversion, with an extremely high order of non-linearity. Lanthanide ion-doped UCNPs have unlocked a new type of targeted super-resolution techniques that work by controlling non-saturated UCL emission with high-order non-linearity under low excitation light intensity (Fig. 9e).
